# Publisher Correction: Personality traits vary in their association with brain activity across situations

**DOI:** 10.1038/s42003-024-07325-9

**Published:** 2024-12-07

**Authors:** Samyogita Hardikar, Brontë McKeown, Adam Turnbull, Ting Xu, Sofie L. Valk, Boris C. Bernhardt, Daniel S. Margulies, Michael P. Milham, Elizabeth Jefferies, Robert Leech, Arno Villringer, Jonathan Smallwood

**Affiliations:** 1 Department of Neurology, Max Planck Institute for Human Cognitive and Brain Sciences, Leipzig, Germany; 2 Max Planck School of Cognition, Stephanstrasse 1A, Leipzig, Germany; 3 Department of Psychology, Queens University, Kingston, ON Canada; 4 Department of Psychiatry and Behavioral Sciences, Stanford University, Stanford, CA USA; 5 Department of Brain and Cognitive Sciences, University of Rochester, Rochester, NY USA; 6 Center for the Developing Brain, Child Mind Institute, New York, NY USA; 7 Otto Hahn Group Cognitive Neurogenetics, Max Planck Institute for Human Cognitive and Brain Sciences, Leipzig, Germany; 8 Institute of Neuroscience and Medicine (INM-7: Brain and Behaviour), Research Centre Jülich, Jülich, Germany; 9 Institute of Systems Neuroscience, Heinrich Heine University Düsseldorf, Düsseldorf, Germany; 10 McConnell Brain Imaging Centre, Montreal Neurological Institute and Hospital, McGill University, Montreal, Canada; 11 Integrative Neuroscience and Cognition Center, Centre National de la Recherche Scientifique (CNRS) and Université de Paris, Paris, France; 12 Wellcome Centre for Integrative Neuroimaging, Nuffield Department of Clinical Neurosciences, University of Oxford, Oxford, UK; 13 Department of Psychology, University of York, York, UK; 14 Centre for Neuroimaging Science, King’s College London, London, UK; 15 Day Clinic of Cognitive Neurology, Universitätsklinikum Leipzig, Leipzig, Germany; 16 MindBrainBody Institute, Berlin School of Mind and Brain, Humboldt-Universität zu Berlin, Berlin, Germany; 17 Center for Stroke Research Berlin (CSB), Charité - Universitätsmedizin Berlin, Berlin, Germany

**Keywords:** Personality, Cognitive neuroscience

**Correction to:** *Communications Biology* (2024) **7**:1498; 10.1038/s42003-024-07061-0; Article published online 12 Nov 2024

The original version of this Article contained an error in Figure 2 in which one of the red asterisks was not rendered properly during production. The correct version of Fig. 2 is:
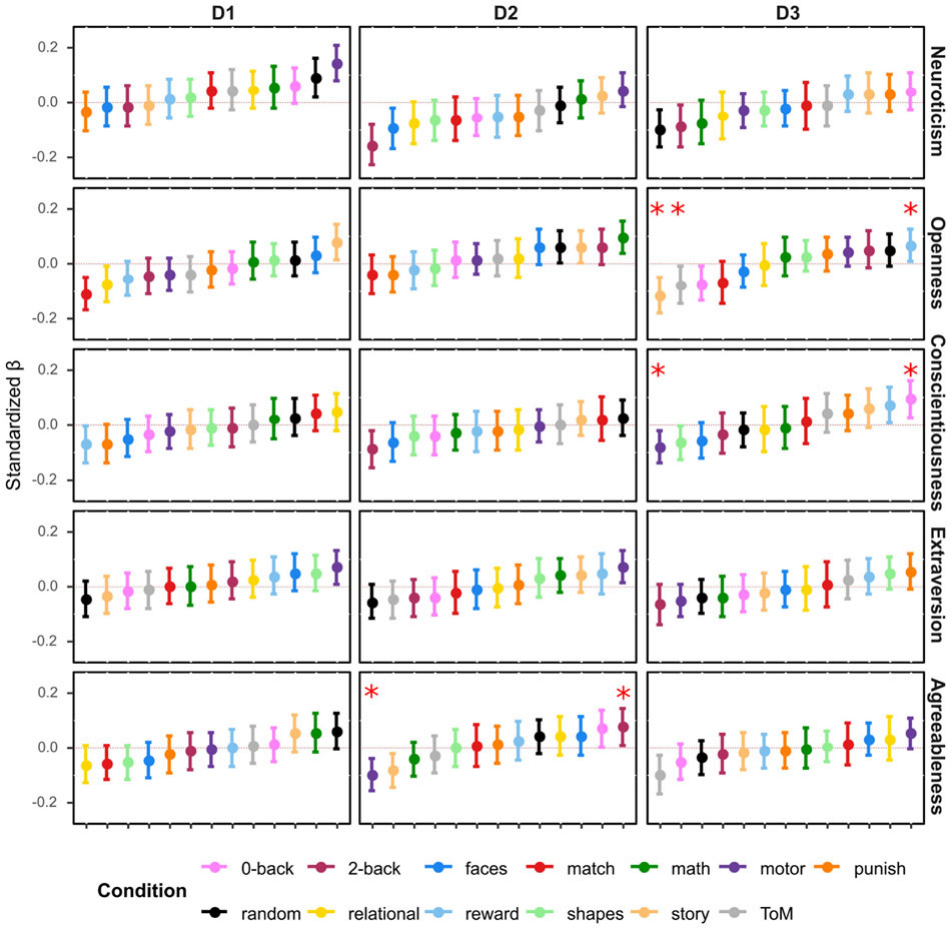


which replaces the previous incorrect version:
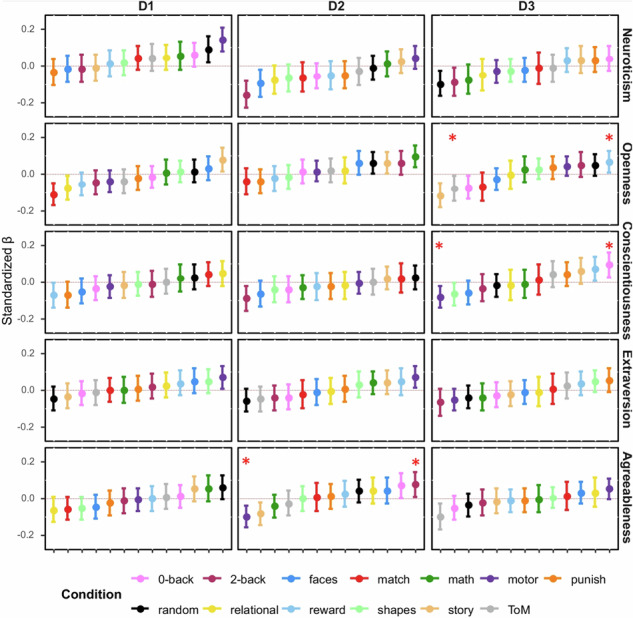


The error/errors have been corrected in both the PDF and HTML versions of the Article.

