# Supplementary material for: Personality traits vary in their association with brain activity across situations
Source: Commun Biol. 2024 Nov 12;7:1498. doi: 10.1038/s42003-024-07061-0 (PMC11557894; doi:10.1038/s42003-024-07061-0)
Supplement: Supplementary file 1 — Supplementary Information [file 42003_2024_7061_MOESM1_ESM.pdf]

Supplementary Information

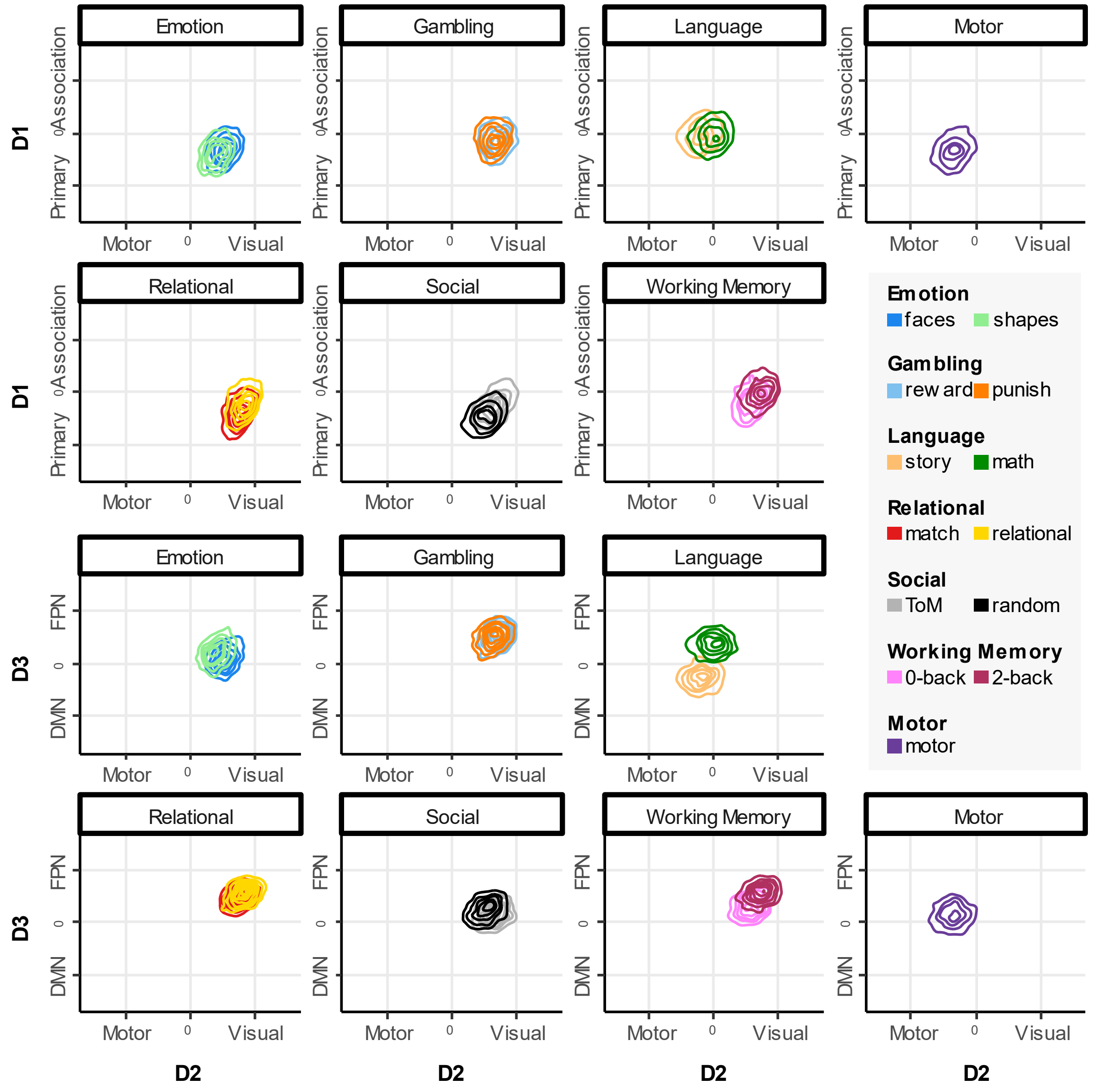

Supplementary Figure 1. Contour plots illustrating the distribution of all individuals' data for each task condition within the state space. Each contour plot, or 2D density plot, shows the distribution of brain maps from the HCP data along two dimensions of brain variation. The location along each dimension is determined by the correlation between the task-maps of each individual and the respective functional connectivity gradient identified at rest (see

Figure 1). Contours depict the distribution of all individuals in this space. Conditions from the same task are plotted in the same panel.

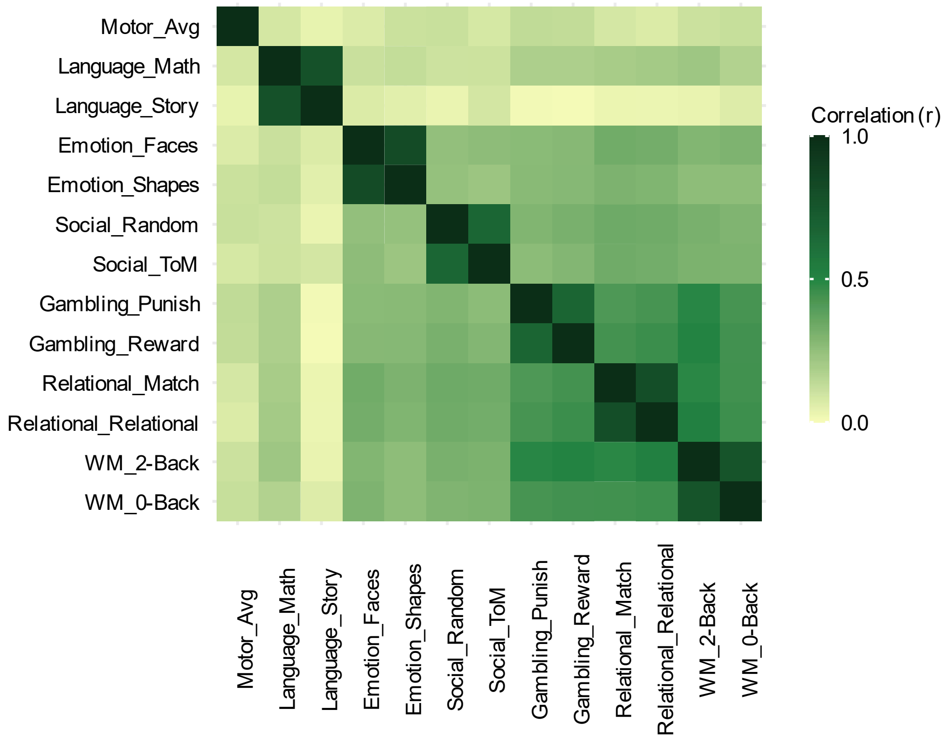

Supplementary Figure 2. **Heatmap showing the group-averaged spearman rank correlations between brain-wide activity patterns across different task conditions.** The group level correlation matrix was derived by first calculating all pair-wise spearman rank correlations between task maps for each individual, and then calculating the mean score across all individuals for each pair at the group level.

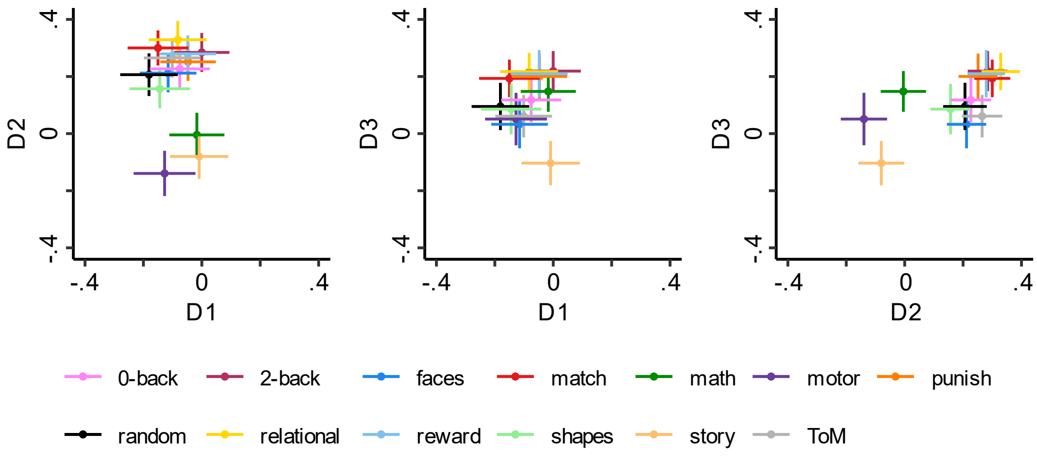

Supplementary Figure 3. Location of whole brain maps in the state space, shown as group means and standard deviations for each task condition.

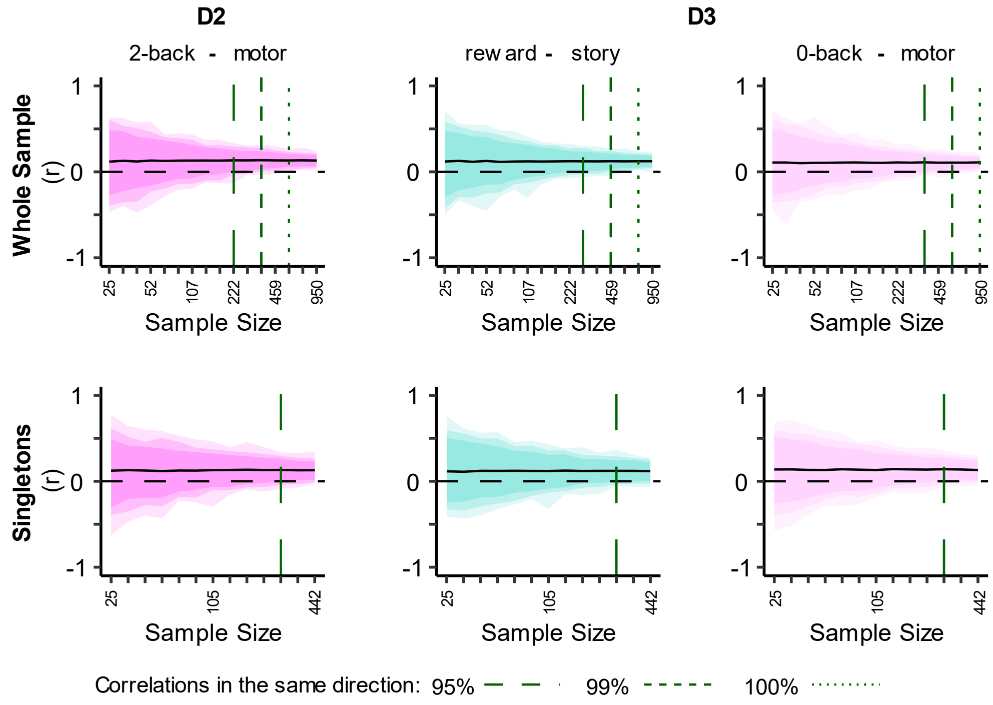

Supplementary Figure 4. Replication of strongest trait-brain associations in a "singletons only" subsample of HCP. These plots summarize the results of a bootstrapping analysis showing the distribution of the same correlations as a function of sample size. Vertical lines

indicate the sample sizes required to consistently find effects in the same direction within the 95%, and 99% confidence intervals, and in the whole range (100%).

Supplementary Table 1. Number of subjects with minimally pre-processed contrast-maps available for each task in the HCP task battery.

**variable**	**(available) n**
Subject	1088
Neuroticism	1083
Openness	1083
Conscientiousness	1083
Extraversion	1083
Agreeableness	1083
Age	1088
Gender	1088
Emotion	1017
Gambling	1060
Language	941
Motor	1032
Relational	994
Theory of Mind (ToM)	1032
Working Memory	1061

Supplementary Table 2. Results of Linear Mixed Models showing the associations between personality traits, task context, and dimensions of brain variation.

DV	IV	Sum Sq	Mean Sq	NumDF	DenDF	F value	Pr(>F)
D1	cond	0.48	0.04	12.0000	11963.65	5.92	0.000000
	Age	0.15	0.15	1.0000	1034.21	22.05	0.000003
	Gender	0.38	0.38	1.0000	1046.55	56.48	0.000000
	motion	0.30	0.30	1.0000	4486.02	44.59	0.000000
	Neuroticism	0.02	0.02	1.0000	1031.87	2.59	0.107586
	Openness	0.01	0.01	1.0000	1043.52	1.37	0.241949
	Conscientiousness	0.00	0.00	1.0000	1042.08	0.41	0.521734
	Extraversion	0.00	0.00	1.0000	1035.67	0.32	0.571320
	Agreeableness	0.00	0.00	1.0000	1012.51	0.13	0.716277
	cond:Neuroticism	0.23	0.02	12.0000	11952.38	2.86	0.000614
	cond:Openness	0.27	0.02	12.0000	11951.15	3.36	0.000066
	cond:Conscientiousness	0.14	0.01	12.0000	11955.93	1.67	0.066138
	cond:Extraversion	0.12	0.01	12.0000	11947.43	1.52	0.109945
	cond:Agreeableness	0.18	0.02	12.0000	11949.54	2.24	0.008204
D2	cond	2.24	0.19	12.0000	11972.46	51.05	0.000000
	Age	0.00	0.00	1.0000	1026.75	1.35	0.244854
	Gender	0.00	0.00	1.0000	1051.29	0.67	0.411903
	motion	0.12	0.12	1.0000	3964.27	33.09	0.000000
	Neuroticism	0.02	0.02	1.0000	1042.05	4.54	0.033380
	Openness	0.01	0.01	1.0000	1041.96	1.63	0.202610
	Conscientiousness	0.00	0.00	1.0000	1052.46	1.17	0.279410
	Extraversion	0.00	0.00	1.0000	1043.06	0.01	0.929634
	Agreeableness	0.00	0.00	1.0000	1027.54	0.00	0.954479
	cond:Neuroticism	0.12	0.01	12.0000	11960.58	2.77	0.000892
	cond:Openness	0.11	0.01	12.0000	11959.69	2.48	0.003106
	cond:Conscientiousness	0.05	0.00	12.0000	11964.77	1.20	0.276057
	cond:Extraversion	0.09	0.01	12.0000	11955.57	2.16	0.011168
	cond:Agreeableness	0.17	0.01	12.0000	11957.55	3.82	0.000008
D3	cond	0.61	0.05	12.0000	11991.89	10.83	0.000000
	Age	0.00	0.00	1.0000	1048.42	0.35	0.554338
	Gender	0.09	0.09	1.0000	1061.92	19.40	0.000012
	motion	0.65	0.65	1.0000	3459.66	138.19	0.000000
	Neuroticism	0.01	0.01	1.0000	1048.19	1.38	0.240672
	Openness	0.00	0.00	1.0000	1058.04	0.11	0.735335

Conscientiousness	0.00	0.00	1.0000	1060.99	0.00	0.996172
Extraversion	0.00	0.00	1.0000	1051.58	0.18	0.670017
Agreeableness	0.00	0.00	1.0000	1029.67	0.30	0.585759
cond:Neuroticism	0.12	0.01	12.0000	11979.31	2.04	0.017536
cond:Openness	0.25	0.02	12.0000	11978.93	4.40	0.000001
cond:Conscientiousness	0.20	0.02	12.0000	11984.30	3.57	0.000025
cond:Extraversion	0.10	0.01	12.0000	11974.14	1.70	0.059102
cond:Agreeableness	0.10	0.01	12.0000	11976.01	1.78	0.045507

DV= Dependent Variable; **IV**= Independent Variable; **cond**=condition

Supplementary Table 3. Estimated marginal means of all task contrast maps along state-space dimensions.

Dimension	condition	emmean	SE	df	lower CL	upper CL
D1	0back	-0.074	0.0032	2053.36	-0.080	-0.068
	2back	0.002	0.0032	2053.36	-0.005	0.008
	faces	-0.113	0.0033	2150.67	-0.120	-0.107
	match	-0.149	0.0033	2216.20	-0.156	-0.143
	math	-0.015	0.0034	2382.98	-0.022	-0.009
	motor	-0.128	0.0033	2134.89	-0.135	-0.122
	punish	-0.045	0.0032	2060.85	-0.051	-0.038
	random	-0.180	0.0033	2117.63	-0.186	-0.173
	relational	-0.082	0.0033	2216.20	-0.088	-0.075
	reward	-0.046	0.0032	2060.85	-0.052	-0.039
	shapes	-0.142	0.0033	2150.67	-0.148	-0.135
	story	-0.008	0.0034	2382.98	-0.014	-0.001
	ToM	-0.100	0.0033	2117.63	-0.106	-0.094
D2	0back	0.227	0.0023	2533.41	0.222	0.231
	2back	0.284	0.0023	2533.41	0.280	0.289
	faces	0.211	0.0023	2655.56	0.207	0.216
	match	0.300	0.0023	2739.28	0.295	0.304
	math	-0.005	0.0024	2953.96	-0.010	0.000
	motor	-0.139	0.0023	2635.63	-0.144	-0.135
	punish	0.251	0.0023	2542.29	0.247	0.256
	random	0.206	0.0023	2613.97	0.201	0.210
	relational	0.328	0.0023	2739.28	0.324	0.333
	reward	0.279	0.0023	2542.29	0.275	0.284
	shapes	0.157	0.0023	2655.56	0.152	0.161
	story	-0.080	0.0024	2953.96	-0.085	-0.076
	ToM	0.264	0.0023	2613.97	0.260	0.269
D3	0back	0.117	0.0025	3108.27	0.112	0.122
	2back	0.218	0.0025	3108.27	0.213	0.223
	faces	0.032	0.0026	3254.83	0.027	0.037
	match	0.194	0.0026	3355.92	0.189	0.199
	math	0.147	0.0026	3616.68	0.142	0.153
	motor	0.053	0.0026	3229.93	0.048	0.058
	punish	0.199	0.0025	3118.63	0.194	0.204

random	0.094	0.0026	3204.54	0.089	0.099
relational	0.218	0.0026	3355.92	0.213	0.223
reward	0.209	0.0025	3118.63	0.204	0.214
shapes	0.085	0.0026	3254.83	0.080	0.090
story	-0.104	0.0026	3616.68	-0.109	-0.098
ToM	0.060	0.0026	3204.54	0.055	0.065

Supplementary Table 4. Estimated marginal trends (slopes) of personality traits for all task conditions along D1 (primary- association)

condition	$\beta_{Neuroticism}$	SE	df	CL $_{lower}$	CL $_{upper}$	t.ratio	p.value
0back	0.0008	0.0005	6739.25	-0.0001	0.0017	1.731	0.08358
2back	-0.0002	0.0005	6739.24	-0.0011	0.0007	-0.419	0.67548
faces	-0.0002	0.0005	6929.58	-0.0011	0.0007	-0.457	0.64775
match	0.0006	0.0005	7041.75	-0.0004	0.0015	1.208	0.22696
math	0.0007	0.0005	7415.50	-0.0003	0.0016	1.366	0.17203
motor	0.0020	0.0005	6840.75	0.0011	0.0030	4.322	0.00002
punish	-0.0005	0.0005	6740.19	-0.0014	0.0004	-1.032	0.30188
random	0.0012	0.0005	6872.28	0.0002	0.0021	2.479	0.01321
relational	0.0006	0.0005	7041.75	-0.0004	0.0015	1.226	0.22031
reward	0.0001	0.0005	6740.19	-0.0008	0.0011	0.280	0.77982
shapes	0.0002	0.0005	6929.58	-0.0007	0.0012	0.464	0.64235
story	-0.0001	0.0005	7415.50	-0.0011	0.0008	-0.306	0.75964
ToM	0.0006	0.0005	6872.28	-0.0004	0.0015	1.206	0.22787
**condition**	**$\beta_{Openness}$**	**SE**	**df**	**CL $_{lower}$**	**CL $_{upper}$**	**t.ratio**	**p.value**
0back	-0.0003	0.0005	6615.40	-0.0013	0.0007	-0.602	0.54746
2back	-0.0007	0.0005	6615.39	-0.0017	0.0002	-1.460	0.14434
faces	0.0005	0.0005	6820.61	-0.0005	0.0015	0.932	0.35147
match	-0.0019	0.0005	6943.89	-0.0029	-0.0008	-3.623	0.00029
math	0.0001	0.0005	7361.77	-0.0009	0.0012	0.244	0.80740
motor	-0.0007	0.0005	6746.85	-0.0017	0.0003	-1.413	0.15776
punish	-0.0003	0.0005	6549.14	-0.0013	0.0006	-0.672	0.50138
random	0.0002	0.0005	6748.70	-0.0008	0.0012	0.418	0.67572
relational	-0.0012	0.0005	6943.89	-0.0022	-0.0002	-2.425	0.01534
reward	-0.0009	0.0005	6549.14	-0.0019	0.0001	-1.768	0.07705
shapes	0.0002	0.0005	6820.61	-0.0008	0.0012	0.311	0.75557
story	0.0012	0.0005	7361.77	0.0002	0.0023	2.360	0.01830
ToM	-0.0006	0.0005	6748.70	-0.0016	0.0003	-1.282	0.19999
**condition**	**$\beta_{Conscientiousness}$**	**SE**	**df**	**CL $_{lower}$**	**CL $_{upper}$**	**t.ratio**	**p.value**
0back	-0.0006	0.0006	6917.68	-0.0017	0.0005	-1.049	0.29401
2back	-0.0002	0.0006	6917.67	-0.0013	0.0009	-0.307	0.75855
faces	-0.0008	0.0006	7103.86	-0.0020	0.0003	-1.461	0.14399
match	0.0007	0.0006	7247.03	-0.0004	0.0018	1.185	0.23615
math	0.0003	0.0006	7625.04	-0.0008	0.0015	0.549	0.58297
motor	-0.0005	0.0006	7054.14	-0.0016	0.0007	-0.797	0.42538

punish	-0.0011	0.0006	6840.30	-0.0022	0.0000	-1.997	0.04588
random	0.0004	0.0006	7112.79	-0.0007	0.0015	0.685	0.49334
relational	0.0007	0.0006	7247.03	-0.0004	0.0019	1.294	0.19569
reward	-0.0012	0.0006	6840.30	-0.0023	-0.0001	-2.107	0.03516
shapes	-0.0003	0.0006	7103.86	-0.0014	0.0009	-0.439	0.66033
story	-0.0003	0.0006	7625.04	-0.0014	0.0009	-0.481	0.63086
ToM	0.0000	0.0006	7112.79	-0.0011	0.0011	0.042	0.96611

**condition**	**$\beta_{Extraversion}$**	**SE**	**df**	**CL $_{lower}$**	**CL $_{upper}$**	**t.ratio**	**p.value**
0back	-0.0003	0.0006	6898.90	-0.0014	0.0008	-0.538	0.59030
2back	0.0003	0.0006	6898.89	-0.0008	0.0014	0.561	0.57472
faces	0.0008	0.0006	6993.66	-0.0003	0.0019	1.380	0.16762
match	0.0000	0.0006	7178.98	-0.0011	0.0011	-0.046	0.96293
math	0.0000	0.0006	7455.71	-0.0011	0.0011	0.000	0.99963
motor	0.0012	0.0006	7066.46	0.0001	0.0023	2.201	0.02778
punish	0.0001	0.0006	6873.23	-0.0010	0.0012	0.181	0.85642
random	-0.0008	0.0006	6959.69	-0.0019	0.0003	-1.421	0.15527
relational	0.0004	0.0006	7178.98	-0.0007	0.0015	0.757	0.44892
reward	0.0006	0.0006	6873.23	-0.0005	0.0017	1.104	0.26967
shapes	0.0008	0.0006	6993.66	-0.0003	0.0019	1.509	0.13142
story	-0.0006	0.0006	7455.71	-0.0017	0.0005	-1.011	0.31212
ToM	-0.0002	0.0006	6959.69	-0.0013	0.0009	-0.421	0.67371

**condition**	**$\beta_{Agreeableness}$**	**SE**	**df**	**CL $_{lower}$**	**CL $_{upper}$**	**t.ratio**	**p.value**
0back	0.0002	0.0006	6534.58	-0.0009	0.0013	0.303	0.76220
2back	-0.0002	0.0006	6534.57	-0.0013	0.0009	-0.369	0.71241
faces	-0.0008	0.0006	6658.90	-0.0019	0.0003	-1.357	0.17494
match	-0.0010	0.0006	6774.67	-0.0021	0.0001	-1.814	0.06973
math	0.0009	0.0006	7336.52	-0.0003	0.0020	1.444	0.14888
motor	-0.0001	0.0006	6683.68	-0.0012	0.0010	-0.220	0.82589
punish	-0.0004	0.0006	6544.37	-0.0015	0.0007	-0.748	0.45459
random	0.0010	0.0006	6585.19	-0.0001	0.0021	1.809	0.07056
relational	-0.0011	0.0006	6774.67	-0.0022	0.0001	-1.861	0.06278
reward	0.0000	0.0006	6544.37	-0.0011	0.0011	-0.076	0.93924
shapes	-0.0010	0.0006	6658.90	-0.0021	0.0001	-1.733	0.08309
story	0.0009	0.0006	7336.52	-0.0003	0.0020	1.512	0.13070
ToM	0.0001	0.0006	6585.19	-0.0010	0.0013	0.263	0.79247

Supplementary Table 5. Estimated marginal trends (slopes) of personality traits for all task conditions along D2 (motor- visual)

condition	$\beta_{Neuroticism}$	SE	df	CL $_{lower}$	CL $_{upper}$	t.ratio	p.value
0back	-0.0005	0.0003	7616.59	-0.0012	0.0001	-1.570	0.11656
2back	-0.0014	0.0003	7616.58	-0.0021	-0.0008	-4.291	0.00002
faces	-0.0009	0.0003	7807.57	-0.0015	-0.0002	-2.546	0.01092
match	-0.0005	0.0003	7923.02	-0.0012	0.0002	-1.503	0.13281
math	0.0001	0.0004	8300.29	-0.0006	0.0008	0.273	0.78508
motor	0.0004	0.0003	7722.01	-0.0002	0.0011	1.305	0.19178
punish	-0.0005	0.0003	7617.66	-0.0011	0.0002	-1.384	0.16644
random	-0.0001	0.0003	7749.94	-0.0008	0.0005	-0.368	0.71276
relational	-0.0007	0.0003	7923.02	-0.0013	0.0000	-1.942	0.05211
reward	-0.0005	0.0003	7617.66	-0.0011	0.0002	-1.365	0.17226
shapes	-0.0006	0.0003	7807.57	-0.0013	0.0001	-1.775	0.07596
story	0.0003	0.0004	8300.29	-0.0004	0.0010	0.733	0.46384
ToM	-0.0003	0.0003	7749.94	-0.0010	0.0004	-0.876	0.38107
**condition**	$\beta_{Openness}$	**SE**	**df**	**CL $_{lower}$**	**CL $_{upper}$**	**t.ratio**	**p.value**
0back	0.0001	0.0004	7459.87	-0.0006	0.0008	0.382	0.70238
2back	0.0007	0.0004	7459.86	0.0000	0.0014	1.846	0.06487
faces	0.0006	0.0004	7672.47	-0.0001	0.0013	1.723	0.08488
match	-0.0004	0.0004	7798.44	-0.0011	0.0003	-1.135	0.25624
math	0.0012	0.0004	8225.09	0.0004	0.0019	3.152	0.00163
motor	0.0002	0.0004	7599.32	-0.0005	0.0009	0.490	0.62435
punish	-0.0005	0.0004	7388.06	-0.0012	0.0002	-1.265	0.20591
random	0.0007	0.0004	7600.45	0.0000	0.0014	1.941	0.05234
relational	0.0002	0.0004	7798.44	-0.0005	0.0009	0.504	0.61404
reward	-0.0002	0.0004	7388.06	-0.0009	0.0005	-0.689	0.49091
shapes	-0.0002	0.0004	7672.47	-0.0009	0.0005	-0.457	0.64736
story	0.0007	0.0004	8225.09	0.0000	0.0015	1.983	0.04741
ToM	0.0002	0.0004	7600.45	-0.0005	0.0009	0.525	0.59938
**condition**	$\beta_{Conscientiousness}$	**SE**	**df**	**CL $_{lower}$**	**CL $_{upper}$**	**t.ratio**	**p.value**
0back	-0.0005	0.0004	7805.43	-0.0013	0.0003	-1.178	0.23899
2back	-0.0010	0.0004	7805.40	-0.0018	-0.0002	-2.568	0.01024
faces	-0.0007	0.0004	7988.24	-0.0015	0.0001	-1.760	0.07842
match	0.0002	0.0004	8132.40	-0.0006	0.0010	0.504	0.61448
math	-0.0004	0.0004	8510.75	-0.0012	0.0004	-0.926	0.35463
motor	-0.0001	0.0004	7943.36	-0.0009	0.0007	-0.206	0.83699

punish	-0.0002	0.0004	7727.19	-0.0010	0.0005	-0.614	0.53926
random	0.0003	0.0004	7999.99	-0.0005	0.0011	0.789	0.43029
relational	-0.0002	0.0004	8132.40	-0.0010	0.0006	-0.519	0.60390
reward	-0.0003	0.0004	7727.19	-0.0011	0.0005	-0.635	0.52554
shapes	-0.0005	0.0004	7988.24	-0.0013	0.0003	-1.175	0.24014
story	0.0003	0.0004	8510.75	-0.0006	0.0011	0.626	0.53124
ToM	0.0000	0.0004	7999.99	-0.0008	0.0008	-0.057	0.95491
**condition**	**$\beta_{Extraversion}$**	**SE**	**df**	**CL $_{lower}$**	**CL $_{upper}$**	**t.ratio**	**p.value**
0back	-0.0005	0.0004	7784.25	-0.0012	0.0003	-1.162	0.24543
2back	-0.0005	0.0004	7784.24	-0.0013	0.0003	-1.253	0.21008
faces	-0.0001	0.0004	7880.49	-0.0009	0.0007	-0.329	0.74185
match	-0.0003	0.0004	8062.57	-0.0011	0.0005	-0.637	0.52439
math	0.0005	0.0004	8341.40	-0.0003	0.0013	1.214	0.22475
motor	0.0010	0.0004	7952.31	0.0002	0.0018	2.422	0.01545
punish	0.0001	0.0004	7755.99	-0.0007	0.0009	0.201	0.84080
random	-0.0007	0.0004	7843.34	-0.0015	0.0001	-1.757	0.07889
relational	-0.0001	0.0004	8062.57	-0.0009	0.0007	-0.167	0.86749
reward	0.0005	0.0004	7755.99	-0.0003	0.0013	1.209	0.22672
shapes	0.0003	0.0004	7880.49	-0.0004	0.0011	0.866	0.38649
story	0.0005	0.0004	8341.40	-0.0003	0.0013	1.312	0.18956
ToM	-0.0005	0.0004	7843.34	-0.0013	0.0002	-1.378	0.16811
**condition**	**$\beta_{Agreeableness}$**	**SE**	**df**	**CL $_{lower}$**	**CL $_{upper}$**	**t.ratio**	**p.value**
0back	0.0008	0.0004	7410.17	0.0000	0.0016	2.047	0.04069
2back	0.0009	0.0004	7410.16	0.0001	0.0017	2.173	0.02981
faces	0.0005	0.0004	7536.37	-0.0003	0.0013	1.211	0.22587
match	0.0001	0.0004	7656.48	-0.0007	0.0009	0.165	0.86896
math	-0.0006	0.0004	8227.56	-0.0014	0.0003	-1.354	0.17572
motor	-0.0014	0.0004	7563.87	-0.0022	-0.0006	-3.345	0.00083
punish	0.0001	0.0004	7417.64	-0.0007	0.0009	0.301	0.76351
random	0.0005	0.0004	7459.81	-0.0003	0.0013	1.255	0.20966
relational	0.0005	0.0004	7656.48	-0.0003	0.0013	1.119	0.26299
reward	0.0002	0.0004	7417.64	-0.0006	0.0010	0.595	0.55188
shapes	0.0000	0.0004	7536.37	-0.0008	0.0008	-0.026	0.97905
story	-0.0012	0.0004	8227.56	-0.0020	-0.0003	-2.713	0.00669
ToM	-0.0003	0.0004	7459.81	-0.0011	0.0005	-0.796	0.42618

Supplementary Table 6. Estimated marginal trends (slopes) of personality traits for all task conditions along D3 (DMN- FPN)

condition	$\beta_{Neuroticism}$	SE	df	CL lower	CL upper	t.ratio	p.value
0back	0.0004	0.0004	8724.24	-0.0003	0.0011	1.069	0.28532
2back	-0.0008	0.0004	8724.23	-0.0016	-0.0001	-2.217	0.02663
faces	-0.0003	0.0004	8897.12	-0.0010	0.0005	-0.734	0.46312
match	-0.0001	0.0004	9007.30	-0.0009	0.0006	-0.315	0.75295
math	-0.0007	0.0004	9367.33	-0.0015	0.0000	-1.847	0.06475
motor	-0.0004	0.0004	8826.35	-0.0011	0.0003	-1.075	0.28227
punish	0.0003	0.0004	8727.02	-0.0004	0.0011	0.905	0.36544
random	-0.0011	0.0004	8846.86	-0.0018	-0.0004	-2.914	0.00357
relational	-0.0004	0.0004	9007.30	-0.0012	0.0003	-1.162	0.24507
reward	0.0003	0.0004	8727.02	-0.0004	0.0011	0.875	0.38140
shapes	-0.0003	0.0004	8897.12	-0.0011	0.0004	-0.844	0.39858
story	0.0003	0.0004	9367.33	-0.0005	0.0011	0.795	0.42683
ToM	-0.0001	0.0004	8846.86	-0.0009	0.0006	-0.336	0.73677
**condition**	$\beta_{Openness}$	**SE**	**df**	**CL lower**	**CL upper**	**t.ratio**	**p.value**
0back	-0.0009	0.0004	8587.97	-0.0017	-0.0002	-2.361	0.01824
2back	0.0005	0.0004	8587.96	-0.0002	0.0013	1.380	0.16777
faces	-0.0004	0.0004	8788.00	-0.0012	0.0004	-1.006	0.31437
match	-0.0007	0.0004	8904.52	-0.0015	0.0001	-1.834	0.06673
math	0.0003	0.0004	9315.51	-0.0006	0.0011	0.619	0.53613
motor	0.0006	0.0004	8720.51	-0.0002	0.0014	1.491	0.13606
punish	0.0004	0.0004	8519.61	-0.0004	0.0012	1.062	0.28829
random	0.0007	0.0004	8718.10	-0.0001	0.0014	1.626	0.10402
relational	-0.0001	0.0004	8904.52	-0.0008	0.0007	-0.128	0.89781
reward	0.0008	0.0004	8519.61	0.0001	0.0016	2.122	0.03386
shapes	0.0004	0.0004	8788.00	-0.0004	0.0012	0.905	0.36556
story	-0.0015	0.0004	9315.51	-0.0023	-0.0007	-3.598	0.00032
ToM	-0.0010	0.0004	8718.10	-0.0017	-0.0002	-2.375	0.01759
**condition**	$\beta_{Conscientiousness}$	**SE**	**df**	**CL lower**	**CL upper**	**t.ratio**	**p.value**
0back	0.0012	0.0005	8911.74	0.0003	0.0021	2.663	0.00777
2back	-0.0004	0.0005	8911.73	-0.0013	0.0005	-0.881	0.37817
faces	-0.0008	0.0005	9074.25	-0.0017	0.0001	-1.809	0.07043
match	0.0001	0.0005	9213.58	-0.0008	0.0010	0.252	0.80075
math	-0.0001	0.0005	9568.55	-0.0011	0.0008	-0.271	0.78670
motor	-0.0013	0.0005	9044.66	-0.0022	-0.0004	-2.775	0.00553

punish	0.0006	0.0004	8836.47	-0.0003	0.0015	1.288	0.19770
random	-0.0003	0.0005	9090.15	-0.0012	0.0006	-0.620	0.53554
relational	-0.0002	0.0005	9213.58	-0.0011	0.0007	-0.473	0.63597
reward	0.0010	0.0004	8836.47	0.0001	0.0018	2.152	0.03142
shapes	-0.0010	0.0005	9074.25	-0.0019	-0.0001	-2.181	0.02919
story	0.0007	0.0005	9568.55	-0.0002	0.0017	1.572	0.11592
ToM	0.0005	0.0005	9090.15	-0.0004	0.0014	1.087	0.27693
**condition**	**β $_{Extraversion}$**	**SE**	**df**	**CL $_{lower}$**	**CL $_{upper}$**	**t.ratio**	**p.value**
0back	-0.0004	0.0004	8895.53	-0.0012	0.0005	-0.820	0.41205
2back	-0.0008	0.0004	8895.53	-0.0016	0.0001	-1.773	0.07621
faces	-0.0002	0.0004	8979.95	-0.0010	0.0007	-0.352	0.72459
match	0.0001	0.0004	9156.22	-0.0008	0.0009	0.158	0.87407
math	-0.0005	0.0005	9418.43	-0.0014	0.0004	-0.999	0.31763
motor	-0.0008	0.0004	9057.09	-0.0017	0.0001	-1.811	0.07023
punish	0.0007	0.0004	8868.54	-0.0001	0.0016	1.637	0.10169
random	-0.0006	0.0004	8946.15	-0.0014	0.0003	-1.246	0.21278
relational	-0.0001	0.0004	9156.22	-0.0010	0.0008	-0.249	0.80353
reward	0.0005	0.0004	8868.54	-0.0004	0.0013	1.111	0.26670
shapes	0.0007	0.0004	8979.95	-0.0002	0.0016	1.554	0.12014
story	-0.0003	0.0005	9418.43	-0.0012	0.0006	-0.599	0.54897
ToM	0.0003	0.0004	8946.15	-0.0006	0.0011	0.614	0.53893
**condition**	**β $_{Agreeableness}$**	**SE**	**df**	**CL $_{lower}$**	**CL $_{upper}$**	**t.ratio**	**p.value**
0back	-0.0007	0.0004	8507.89	-0.0016	0.0002	-1.601	0.10938
2back	-0.0003	0.0004	8507.88	-0.0012	0.0006	-0.670	0.50316
faces	0.0004	0.0005	8622.84	-0.0004	0.0013	0.963	0.33580
match	0.0001	0.0005	8740.11	-0.0008	0.0010	0.307	0.75913
math	-0.0001	0.0005	9288.07	-0.0010	0.0009	-0.128	0.89810
motor	0.0008	0.0004	8660.37	-0.0001	0.0017	1.840	0.06580
punish	-0.0002	0.0004	8511.83	-0.0010	0.0007	-0.374	0.70804
random	-0.0005	0.0004	8548.72	-0.0014	0.0003	-1.194	0.23238
relational	0.0003	0.0005	8740.11	-0.0006	0.0012	0.743	0.45733
reward	-0.0002	0.0004	8511.83	-0.0011	0.0007	-0.402	0.68801
shapes	0.0000	0.0005	8622.84	-0.0008	0.0009	0.105	0.91665
story	-0.0002	0.0005	9288.07	-0.0011	0.0007	-0.462	0.64402
ToM	-0.0013	0.0004	8548.72	-0.0021	-0.0004	-2.840	0.00452

Supplementary Table 7.1 Neuroticism* task condition interactions along D1 (primary-association)

contrast	β	SE	df	CL_{lower}	CL_{upper}	t.ratio	p.value
0back - 2back	0.0010	0.0006	11916.92	-0.0001	0.0021	1.80	0.07253
0back - faces	0.0010	0.0006	11962.92	-0.0001	0.0021	1.81	0.06980
0back - match	0.0002	0.0006	11965.96	-0.0009	0.0014	0.41	0.68311
0back - math	0.0001	0.0006	11973.37	-0.0010	0.0013	0.25	0.80598
0back - motor	-0.0012	0.0006	11933.72	-0.0023	-0.0001	-2.18	0.02954
0back - punish	0.0013	0.0006	11936.66	0.0002	0.0024	2.31	0.02109
0back - random	-0.0004	0.0006	11969.37	-0.0015	0.0007	-0.64	0.52261
0back - relational	0.0002	0.0006	11965.96	-0.0009	0.0013	0.39	0.69392
0back - reward	0.0007	0.0006	11936.66	-0.0004	0.0018	1.21	0.22574
0back - shapes	0.0006	0.0006	11962.92	-0.0005	0.0017	1.04	0.29787
0back - story	0.0010	0.0006	11973.37	-0.0002	0.0021	1.66	0.09702
0back - ToM	0.0002	0.0006	11969.37	-0.0009	0.0013	0.43	0.67053
2back - faces	0.0000	0.0006	11962.92	-0.0011	0.0011	0.04	0.97051
2back - match	-0.0008	0.0006	11965.96	-0.0019	0.0003	-1.36	0.17410
2back - math	-0.0009	0.0006	11973.37	-0.0020	0.0003	-1.49	0.13517
2back - motor	-0.0022	0.0006	11933.73	-0.0033	-0.0011	-3.96	0.00007
2back - punish	0.0003	0.0006	11936.66	-0.0008	0.0014	0.51	0.60846
2back - random	-0.0014	0.0006	11969.37	-0.0025	-0.0003	-2.42	0.01550
2back - relational	-0.0008	0.0006	11965.96	-0.0019	0.0003	-1.37	0.16950
2back - reward	-0.0003	0.0006	11936.66	-0.0014	0.0008	-0.58	0.55998
2back - shapes	-0.0004	0.0006	11962.92	-0.0015	0.0007	-0.74	0.46214
2back - story	0.0000	0.0006	11973.37	-0.0012	0.0011	-0.08	0.93615
2back - ToM	-0.0008	0.0006	11969.37	-0.0019	0.0003	-1.36	0.17514
faces - match	-0.0008	0.0006	11928.41	-0.0019	0.0003	-1.39	0.16551
faces - math	-0.0009	0.0006	11946.63	-0.0020	0.0003	-1.52	0.12861
faces - motor	-0.0023	0.0006	11970.62	-0.0034	-0.0011	-3.96	0.00008
faces - punish	0.0003	0.0006	11961.69	-0.0008	0.0014	0.47	0.63814
faces - random	-0.0014	0.0006	11932.32	-0.0025	-0.0003	-2.44	0.01465
faces - relational	-0.0008	0.0006	11928.41	-0.0019	0.0003	-1.40	0.16111
faces - reward	-0.0003	0.0006	11961.69	-0.0015	0.0008	-0.61	0.53912
faces - shapes	-0.0004	0.0006	11916.92	-0.0016	0.0007	-0.77	0.44275
faces - story	-0.0001	0.0006	11946.63	-0.0012	0.0011	-0.12	0.90805
faces - ToM	-0.0008	0.0006	11932.32	-0.0019	0.0003	-1.38	0.16650
match - math	-0.0001	0.0006	11940.95	-0.0012	0.0011	-0.15	0.87725
match - motor	-0.0015	0.0006	11967.67	-0.0026	-0.0003	-2.55	0.01077
match - punish	0.0011	0.0006	11969.29	-0.0001	0.0022	1.86	0.06238
match - random	-0.0006	0.0006	11938.84	-0.0017	0.0005	-1.04	0.29918

match - relational	0.0000	0.0006	11916.92	-0.0011	0.0011	-0.01	0.98841
match - reward	0.0004	0.0006	11969.29	-0.0007	0.0016	0.79	0.43243
match - shapes	0.0004	0.0006	11928.41	-0.0008	0.0015	0.62	0.53299
match - story	0.0007	0.0006	11940.95	-0.0004	0.0019	1.24	0.21364
match - ToM	0.0000	0.0006	11938.84	-0.0011	0.0011	0.01	0.98897
math - motor	-0.0014	0.0006	11978.19	-0.0025	-0.0002	-2.35	0.01855
math - punish	0.0012	0.0006	11979.96	0.0000	0.0023	1.99	0.04658
math - random	-0.0005	0.0006	11952.01	-0.0016	0.0006	-0.87	0.38636
math - relational	0.0001	0.0006	11940.95	-0.0011	0.0012	0.14	0.88853
math - reward	0.0005	0.0006	11979.96	-0.0006	0.0017	0.93	0.35305
math - shapes	0.0004	0.0006	11946.63	-0.0007	0.0016	0.77	0.44216
math - story	0.0008	0.0006	11916.92	-0.0003	0.0020	1.38	0.16735
math - ToM	0.0001	0.0006	11952.01	-0.0010	0.0012	0.17	0.86569
motor - punish	0.0025	0.0006	11936.29	0.0014	0.0036	4.47	0.00001
motor - random	0.0009	0.0006	11978.50	-0.0002	0.0020	1.52	0.12768
motor - relational	0.0015	0.0006	11967.67	0.0003	0.0026	2.54	0.01123
motor - reward	0.0019	0.0006	11936.29	0.0008	0.0030	3.38	0.00072
motor - shapes	0.0018	0.0006	11970.62	0.0007	0.0029	3.19	0.00142
motor - story	0.0022	0.0006	11978.19	0.0010	0.0033	3.76	0.00017
motor - ToM	0.0015	0.0006	11978.50	0.0004	0.0026	2.58	0.00980
punish - random	-0.0017	0.0006	11968.10	-0.0028	-0.0005	-2.93	0.00340
punish - relational	-0.0011	0.0006	11969.29	-0.0022	0.0000	-1.88	0.06034
punish - reward	-0.0006	0.0006	11916.92	-0.0017	0.0005	-1.10	0.27296
punish - shapes	-0.0007	0.0006	11961.69	-0.0018	0.0004	-1.24	0.21396
punish - story	-0.0003	0.0006	11979.96	-0.0015	0.0008	-0.58	0.56419
punish - ToM	-0.0011	0.0006	11968.10	-0.0022	0.0001	-1.86	0.06223
random - relational	0.0006	0.0006	11938.84	-0.0005	0.0017	1.02	0.30604
random - reward	0.0010	0.0006	11968.10	-0.0001	0.0022	1.84	0.06545
random - shapes	0.0010	0.0006	11932.32	-0.0002	0.0021	1.67	0.09456
random - story	0.0013	0.0006	11952.01	0.0002	0.0025	2.27	0.02297
random - ToM	0.0006	0.0006	11916.92	-0.0005	0.0017	1.06	0.28856
relational - reward	0.0005	0.0006	11969.29	-0.0007	0.0016	0.80	0.42388
relational - shapes	0.0004	0.0006	11928.41	-0.0008	0.0015	0.64	0.52346
relational - story	0.0007	0.0006	11940.95	-0.0004	0.0019	1.26	0.20843
relational - ToM	0.0000	0.0006	11938.84	-0.0011	0.0011	0.03	0.97731
reward - shapes	-0.0001	0.0006	11961.69	-0.0012	0.0010	-0.16	0.87419
reward - story	0.0003	0.0006	11979.96	-0.0009	0.0014	0.48	0.62776
reward - ToM	-0.0004	0.0006	11968.10	-0.0015	0.0007	-0.78	0.43694
shapes - story	0.0004	0.0006	11946.63	-0.0008	0.0015	0.64	0.52504
shapes - ToM	-0.0003	0.0006	11932.32	-0.0015	0.0008	-0.61	0.53899
story - ToM	-0.0007	0.0006	11952.01	-0.0019	0.0004	-1.24	0.21546

Supplementary Table 7.2 Openness* task condition interactions along D1 (primary-association)

contrast	β	SE	df	CL lower	CL upper	t.ratio	p.value
0back - 2back	0.0004	0.0006	11916.92	-0.0007	0.0016	0.72	0.47073
0back - faces	-0.0008	0.0006	11958.88	-0.0020	0.0004	-1.28	0.19882
0back - match	0.0015	0.0006	11963.39	0.0004	0.0027	2.56	0.01035
0back - math	-0.0004	0.0006	11975.61	-0.0016	0.0008	-0.70	0.48675
0back - motor	0.0004	0.0006	11935.74	-0.0008	0.0016	0.69	0.49206
0back - punish	0.0000	0.0006	11933.93	-0.0011	0.0012	0.06	0.95443
0back - random	-0.0005	0.0006	11960.10	-0.0017	0.0007	-0.85	0.39349
0back - relational	0.0009	0.0006	11963.39	-0.0002	0.0021	1.55	0.12074
0back - reward	0.0006	0.0006	11933.93	-0.0006	0.0017	0.98	0.32909
0back - shapes	-0.0005	0.0006	11958.88	-0.0016	0.0007	-0.76	0.44594
0back - story	-0.0015	0.0006	11975.61	-0.0027	-0.0003	-2.50	0.01259
0back - ToM	0.0003	0.0006	11960.10	-0.0008	0.0015	0.58	0.56428
2back - faces	-0.0012	0.0006	11958.89	-0.0024	0.0000	-2.00	0.04570
2back - match	0.0011	0.0006	11963.40	-0.0001	0.0023	1.85	0.06362
2back - math	-0.0009	0.0006	11975.62	-0.0021	0.0003	-1.39	0.16395
2back - motor	0.0000	0.0006	11935.74	-0.0012	0.0012	-0.03	0.97651
2back - punish	-0.0004	0.0006	11933.93	-0.0016	0.0008	-0.67	0.50565
2back - random	-0.0009	0.0006	11960.11	-0.0021	0.0002	-1.57	0.11670
2back - relational	0.0005	0.0006	11963.40	-0.0007	0.0017	0.84	0.39955
2back - reward	0.0002	0.0006	11933.93	-0.0010	0.0013	0.25	0.80012
2back - shapes	-0.0009	0.0006	11958.89	-0.0021	0.0003	-1.48	0.14008
2back - story	-0.0020	0.0006	11975.62	-0.0032	-0.0008	-3.19	0.00142
2back - ToM	-0.0001	0.0006	11960.11	-0.0013	0.0011	-0.14	0.88943
faces - match	0.0023	0.0006	11934.94	0.0011	0.0035	3.81	0.00014
faces - math	0.0003	0.0006	11953.48	-0.0009	0.0016	0.56	0.57882
faces - motor	0.0012	0.0006	11968.42	0.0000	0.0024	1.96	0.05033
faces - punish	0.0008	0.0006	11960.23	-0.0004	0.0020	1.35	0.17852
faces - random	0.0003	0.0006	11926.83	-0.0009	0.0014	0.43	0.66534
faces - relational	0.0017	0.0006	11934.94	0.0005	0.0029	2.81	0.00497
faces - reward	0.0014	0.0006	11960.23	0.0002	0.0025	2.25	0.02415
faces - shapes	0.0003	0.0006	11916.92	-0.0009	0.0015	0.52	0.60346
faces - story	-0.0008	0.0006	11953.48	-0.0020	0.0005	-1.23	0.21788
faces - ToM	0.0011	0.0006	11926.83	-0.0001	0.0023	1.85	0.06399
match - math	-0.0020	0.0006	11943.47	-0.0032	-0.0008	-3.18	0.00150
match - motor	-0.0011	0.0006	11965.65	-0.0023	0.0001	-1.87	0.06097
match - punish	-0.0015	0.0006	11971.11	-0.0027	-0.0003	-2.51	0.01193
match - random	-0.0021	0.0006	11939.24	-0.0033	-0.0009	-3.40	0.00069

match - relational	-0.0006	0.0006	11916.92	-0.0018	0.0006	-1.00	0.31674
match - reward	-0.0010	0.0006	11971.11	-0.0022	0.0002	-1.61	0.10728
match - shapes	-0.0020	0.0006	11934.94	-0.0032	-0.0008	-3.30	0.00097
match - story	-0.0031	0.0006	11943.47	-0.0043	-0.0019	-4.96	0.00000
match - ToM	-0.0012	0.0006	11939.24	-0.0024	0.0000	-1.98	0.04726
math - motor	0.0008	0.0006	11975.05	-0.0004	0.0021	1.36	0.17498
math - punish	0.0005	0.0006	11982.14	-0.0007	0.0017	0.75	0.45179
math - random	-0.0001	0.0006	11952.04	-0.0013	0.0011	-0.13	0.89299
math - relational	0.0014	0.0006	11943.47	0.0001	0.0026	2.19	0.02829
math - reward	0.0010	0.0006	11982.14	-0.0002	0.0022	1.64	0.10102
math - shapes	0.0000	0.0006	11953.48	-0.0012	0.0012	-0.05	0.96144
math - story	-0.0011	0.0006	11916.92	-0.0023	0.0001	-1.75	0.07939
math - ToM	0.0008	0.0006	11952.04	-0.0004	0.0020	1.25	0.21080
motor - punish	-0.0004	0.0006	11936.28	-0.0015	0.0008	-0.63	0.52724
motor - random	-0.0009	0.0006	11967.63	-0.0021	0.0003	-1.53	0.12594
motor - relational	0.0005	0.0006	11965.65	-0.0007	0.0017	0.87	0.38603
motor - reward	0.0002	0.0006	11936.28	-0.0010	0.0013	0.28	0.77852
motor - shapes	-0.0009	0.0006	11968.42	-0.0021	0.0003	-1.44	0.15054
motor - story	-0.0019	0.0006	11975.05	-0.0032	-0.0007	-3.15	0.00165
motor - ToM	-0.0001	0.0006	11967.63	-0.0012	0.0011	-0.11	0.91322
punish - random	-0.0005	0.0006	11955.72	-0.0017	0.0006	-0.91	0.36137
punish - relational	0.0009	0.0006	11971.11	-0.0003	0.0021	1.50	0.13375
punish - reward	0.0005	0.0006	11916.92	-0.0006	0.0017	0.92	0.35636
punish - shapes	-0.0005	0.0006	11960.23	-0.0017	0.0007	-0.82	0.41162
punish - story	-0.0016	0.0006	11982.14	-0.0028	-0.0004	-2.56	0.01057
punish - ToM	0.0003	0.0006	11955.72	-0.0009	0.0015	0.52	0.60196
random - relational	0.0014	0.0006	11939.24	0.0003	0.0026	2.39	0.01699
random - reward	0.0011	0.0006	11955.72	-0.0001	0.0023	1.83	0.06798
random - shapes	0.0001	0.0006	11926.83	-0.0011	0.0012	0.09	0.92977
random - story	-0.0010	0.0006	11952.04	-0.0022	0.0002	-1.66	0.09731
random - ToM	0.0009	0.0006	11916.92	-0.0003	0.0020	1.42	0.15423
relational - reward	-0.0004	0.0006	11971.11	-0.0015	0.0008	-0.60	0.55151
relational - shapes	-0.0014	0.0006	11934.94	-0.0026	-0.0002	-2.29	0.02184
relational - story	-0.0025	0.0006	11943.47	-0.0037	-0.0013	-3.97	0.00007
relational - ToM	-0.0006	0.0006	11939.24	-0.0018	0.0006	-0.98	0.32908
reward - shapes	-0.0010	0.0006	11960.23	-0.0022	0.0001	-1.73	0.08354
reward - story	-0.0021	0.0006	11982.14	-0.0033	-0.0009	-3.44	0.00057
reward - ToM	-0.0002	0.0006	11955.72	-0.0014	0.0009	-0.39	0.69585
shapes - story	-0.0011	0.0006	11953.48	-0.0023	0.0001	-1.74	0.08205
shapes - ToM	0.0008	0.0006	11926.83	-0.0004	0.0020	1.33	0.18299
story - ToM	0.0019	0.0006	11952.04	0.0007	0.0031	3.04	0.00234

Supplementary Table 7.3 Conscientiousness* task condition interactions along D1 (primary-association)

contrast	β	SE	df	CL_{lower}	CL_{upper}	t.ratio	p.value
0back - 2back	-0.0004	0.0007	11916.91	-0.0018	0.0009	-0.62	0.53701
0back - faces	0.0002	0.0007	11970.61	-0.0011	0.0016	0.36	0.72202
0back - match	-0.0013	0.0007	11974.23	-0.0026	0.0001	-1.85	0.06435
0back - math	-0.0009	0.0007	11988.65	-0.0023	0.0005	-1.31	0.19116
0back - motor	-0.0001	0.0007	11929.53	-0.0015	0.0012	-0.20	0.83911
0back - punish	0.0005	0.0007	11936.98	-0.0008	0.0019	0.78	0.43434
0back - random	-0.0010	0.0007	11977.49	-0.0023	0.0004	-1.43	0.15146
0back - relational	-0.0013	0.0007	11974.23	-0.0027	0.0000	-1.94	0.05223
0back - reward	0.0006	0.0007	11936.98	-0.0007	0.0019	0.87	0.38264
0back - shapes	-0.0003	0.0007	11970.61	-0.0017	0.0010	-0.50	0.61930
0back - story	-0.0003	0.0007	11988.65	-0.0017	0.0011	-0.44	0.66008
0back - ToM	-0.0006	0.0007	11977.49	-0.0020	0.0007	-0.90	0.36884
2back - faces	0.0007	0.0007	11970.62	-0.0007	0.0020	0.97	0.33387
2back - match	-0.0009	0.0007	11974.23	-0.0022	0.0005	-1.24	0.21395
2back - math	-0.0005	0.0007	11988.66	-0.0019	0.0009	-0.71	0.47772
2back - motor	0.0003	0.0007	11929.54	-0.0011	0.0016	0.41	0.68139
2back - punish	0.0010	0.0007	11936.99	-0.0004	0.0023	1.40	0.16141
2back - random	-0.0006	0.0007	11977.49	-0.0019	0.0008	-0.82	0.40990
2back - relational	-0.0009	0.0007	11974.23	-0.0023	0.0004	-1.33	0.18213
2back - reward	0.0010	0.0007	11936.99	-0.0003	0.0023	1.49	0.13580
2back - shapes	0.0001	0.0007	11970.62	-0.0013	0.0014	0.11	0.90942
2back - story	0.0001	0.0007	11988.66	-0.0013	0.0015	0.16	0.87491
2back - ToM	-0.0002	0.0007	11977.49	-0.0015	0.0012	-0.29	0.77313
faces - match	-0.0015	0.0007	11927.96	-0.0029	-0.0002	-2.19	0.02846
faces - math	-0.0012	0.0007	11948.56	-0.0026	0.0002	-1.65	0.09984
faces - motor	-0.0004	0.0007	11976.07	-0.0017	0.0010	-0.55	0.57907
faces - punish	0.0003	0.0007	11966.23	-0.0011	0.0016	0.42	0.67646
faces - random	-0.0012	0.0007	11932.89	-0.0026	0.0001	-1.78	0.07516
faces - relational	-0.0016	0.0007	11927.96	-0.0030	-0.0002	-2.28	0.02250
faces - reward	0.0003	0.0007	11966.23	-0.0010	0.0017	0.51	0.61169
faces - shapes	-0.0006	0.0007	11916.92	-0.0019	0.0008	-0.85	0.39643
faces - story	-0.0006	0.0007	11948.56	-0.0019	0.0008	-0.78	0.43348
faces - ToM	-0.0009	0.0007	11932.89	-0.0022	0.0005	-1.25	0.21243
match - math	0.0004	0.0007	11946.87	-0.0010	0.0018	0.51	0.61206
match - motor	0.0011	0.0007	11972.99	-0.0002	0.0025	1.64	0.10087
match - punish	0.0018	0.0007	11972.05	0.0005	0.0032	2.63	0.00867
match - random	0.0003	0.0007	11940.67	-0.0011	0.0017	0.42	0.67411

match - relational	-0.0001	0.0007	11916.92	-0.0014	0.0013	-0.09	0.92789
match - reward	0.0019	0.0007	11972.05	0.0005	0.0032	2.72	0.00663
match - shapes	0.0009	0.0007	11927.96	-0.0004	0.0023	1.35	0.17759
match - story	0.0010	0.0007	11946.87	-0.0004	0.0024	1.37	0.17221
match - ToM	0.0007	0.0007	11940.67	-0.0007	0.0020	0.95	0.34210
math - motor	0.0008	0.0007	11981.54	-0.0006	0.0022	1.10	0.26928
math - punish	0.0015	0.0007	11985.32	0.0001	0.0028	2.07	0.03862
math - random	-0.0001	0.0007	11956.75	-0.0015	0.0013	-0.10	0.92373
math - relational	-0.0004	0.0007	11946.87	-0.0018	0.0010	-0.60	0.55110
math - reward	0.0015	0.0007	11985.32	0.0001	0.0029	2.16	0.03104
math - shapes	0.0006	0.0007	11948.56	-0.0008	0.0020	0.82	0.41419
math - story	0.0006	0.0007	11916.92	-0.0008	0.0020	0.85	0.39668
math - ToM	0.0003	0.0007	11956.75	-0.0011	0.0017	0.43	0.67065
motor - punish	0.0007	0.0007	11941.61	-0.0007	0.0020	0.98	0.32660
motor - random	-0.0008	0.0007	11982.76	-0.0022	0.0005	-1.23	0.22031
motor - relational	-0.0012	0.0007	11972.99	-0.0026	0.0002	-1.73	0.08334
motor - reward	0.0007	0.0007	11941.61	-0.0006	0.0021	1.07	0.28385
motor - shapes	-0.0002	0.0007	11976.07	-0.0016	0.0012	-0.29	0.76938
motor - story	-0.0002	0.0007	11981.54	-0.0016	0.0012	-0.24	0.80945
motor - ToM	-0.0005	0.0007	11982.76	-0.0018	0.0009	-0.69	0.48838
punish - random	-0.0015	0.0007	11966.70	-0.0029	-0.0002	-2.21	0.02687
punish - relational	-0.0019	0.0007	11972.05	-0.0032	-0.0005	-2.72	0.00660
punish - reward	0.0001	0.0007	11916.92	-0.0013	0.0014	0.09	0.92699
punish - shapes	-0.0009	0.0007	11966.23	-0.0022	0.0005	-1.27	0.20313
punish - story	-0.0008	0.0007	11985.32	-0.0022	0.0005	-1.20	0.23081
punish - ToM	-0.0011	0.0007	11966.70	-0.0025	0.0002	-1.68	0.09376
random - relational	-0.0004	0.0007	11940.67	-0.0017	0.0010	-0.51	0.60909
random - reward	0.0016	0.0007	11966.70	0.0002	0.0029	2.30	0.02124
random - shapes	0.0006	0.0007	11932.89	-0.0007	0.0020	0.93	0.35118
random - story	0.0007	0.0007	11956.75	-0.0007	0.0021	0.96	0.33819
random - ToM	0.0004	0.0007	11916.92	-0.0010	0.0017	0.53	0.59397
relational - reward	0.0019	0.0007	11972.05	0.0006	0.0033	2.81	0.00501
relational - shapes	0.0010	0.0007	11927.96	-0.0004	0.0024	1.44	0.15011
relational - story	0.0010	0.0007	11946.87	-0.0004	0.0024	1.45	0.14592
relational - ToM	0.0007	0.0007	11940.67	-0.0006	0.0021	1.04	0.29794
reward - shapes	-0.0009	0.0007	11966.23	-0.0023	0.0004	-1.36	0.17287
reward - story	-0.0009	0.0007	11985.32	-0.0023	0.0005	-1.29	0.19822
reward - ToM	-0.0012	0.0007	11966.70	-0.0026	0.0001	-1.77	0.07736
shapes - story	0.0000	0.0007	11948.56	-0.0014	0.0014	0.05	0.96340
shapes - ToM	-0.0003	0.0007	11932.89	-0.0016	0.0011	-0.40	0.68941
story - ToM	-0.0003	0.0007	11956.75	-0.0017	0.0011	-0.44	0.66228

Supplementary Table 7.4 Extraversion* task condition interactions along D1 (primary-association)

contrast	β	SE	df	CL_{lower}	CL_{upper}	t.ratio	p.value
0back - 2back	-0.0006	0.0007	11916.92	-0.0019	0.0007	-0.92	0.36009
0back - faces	-0.0011	0.0007	11953.73	-0.0024	0.0002	-1.59	0.11079
0back - match	-0.0003	0.0007	11961.95	-0.0016	0.0010	-0.40	0.68690
0back - math	-0.0003	0.0007	11974.25	-0.0016	0.0010	-0.44	0.66266
0back - motor	-0.0015	0.0007	11935.74	-0.0028	-0.0002	-2.28	0.02253
0back - punish	-0.0004	0.0007	11927.35	-0.0017	0.0009	-0.60	0.54930
0back - random	0.0005	0.0007	11955.91	-0.0008	0.0018	0.74	0.46103
0back - relational	-0.0007	0.0007	11961.95	-0.0020	0.0006	-1.08	0.28231
0back - reward	-0.0009	0.0007	11927.35	-0.0022	0.0004	-1.37	0.17188
0back - shapes	-0.0011	0.0007	11953.73	-0.0024	0.0002	-1.70	0.08879
0back - story	0.0003	0.0007	11974.25	-0.0011	0.0016	0.41	0.67967
0back - ToM	-0.0001	0.0007	11955.91	-0.0014	0.0012	-0.10	0.92390
2back - faces	-0.0005	0.0007	11953.73	-0.0018	0.0008	-0.69	0.49309
2back - match	0.0003	0.0007	11961.95	-0.0010	0.0017	0.50	0.61747
2back - math	0.0003	0.0007	11974.25	-0.0010	0.0016	0.46	0.64879
2back - motor	-0.0009	0.0007	11935.74	-0.0022	0.0004	-1.37	0.16966
2back - punish	0.0002	0.0007	11927.35	-0.0011	0.0015	0.32	0.75138
2back - random	0.0011	0.0007	11955.91	-0.0002	0.0024	1.65	0.09936
2back - relational	-0.0001	0.0007	11961.95	-0.0014	0.0012	-0.17	0.86291
2back - reward	-0.0003	0.0007	11927.35	-0.0016	0.0010	-0.45	0.65227
2back - shapes	-0.0005	0.0007	11953.73	-0.0018	0.0008	-0.79	0.42802
2back - story	0.0009	0.0007	11974.25	-0.0004	0.0022	1.30	0.19204
2back - ToM	0.0005	0.0007	11955.91	-0.0008	0.0018	0.82	0.41485
faces - match	0.0008	0.0007	11931.56	-0.0005	0.0021	1.18	0.23936
faces - math	0.0008	0.0007	11950.03	-0.0006	0.0021	1.12	0.26091
faces - motor	-0.0005	0.0007	11960.01	-0.0018	0.0009	-0.69	0.49335
faces - punish	0.0007	0.0007	11951.19	-0.0006	0.0020	1.00	0.31665
faces - random	0.0016	0.0007	11931.81	0.0002	0.0029	2.33	0.01996
faces - relational	0.0003	0.0007	11931.56	-0.0010	0.0017	0.51	0.61266
faces - reward	0.0002	0.0007	11951.19	-0.0011	0.0015	0.24	0.81169
faces - shapes	-0.0001	0.0007	11916.92	-0.0014	0.0012	-0.11	0.91485
faces - story	0.0013	0.0007	11950.03	0.0000	0.0027	1.97	0.04875
faces - ToM	0.0010	0.0007	11931.81	-0.0003	0.0023	1.50	0.13433
match - math	0.0000	0.0007	11940.00	-0.0014	0.0013	-0.04	0.96995
match - motor	-0.0013	0.0007	11954.75	-0.0026	0.0001	-1.85	0.06386
match - punish	-0.0001	0.0007	11959.18	-0.0014	0.0012	-0.19	0.85134
match - random	0.0008	0.0007	11939.45	-0.0006	0.0021	1.13	0.25769

match - relational	-0.0005	0.0007	11916.92	-0.0018	0.0009	-0.67	0.50516
match - reward	-0.0006	0.0007	11959.18	-0.0019	0.0007	-0.94	0.34477
match - shapes	-0.0009	0.0007	11931.56	-0.0022	0.0005	-1.28	0.19961
match - story	0.0006	0.0007	11940.00	-0.0008	0.0019	0.80	0.42148
match - ToM	0.0002	0.0007	11939.45	-0.0011	0.0015	0.31	0.75807
math - motor	-0.0012	0.0007	11969.74	-0.0026	0.0001	-1.79	0.07295
math - punish	-0.0001	0.0007	11971.87	-0.0014	0.0012	-0.15	0.88306
math - random	0.0008	0.0007	11947.55	-0.0005	0.0021	1.16	0.24745
math - relational	-0.0004	0.0007	11940.00	-0.0018	0.0009	-0.62	0.53499
math - reward	-0.0006	0.0007	11971.87	-0.0019	0.0007	-0.90	0.37058
math - shapes	-0.0008	0.0007	11950.03	-0.0022	0.0005	-1.23	0.21906
math - story	0.0006	0.0007	11916.92	-0.0008	0.0019	0.83	0.40437
math - ToM	0.0002	0.0007	11947.55	-0.0011	0.0016	0.34	0.73207
motor - punish	0.0011	0.0007	11931.94	-0.0002	0.0024	1.69	0.09107
motor - random	0.0020	0.0007	11965.26	0.0007	0.0033	3.00	0.00267
motor - relational	0.0008	0.0007	11954.75	-0.0005	0.0021	1.19	0.23598
motor - reward	0.0006	0.0007	11931.94	-0.0007	0.0019	0.93	0.35357
motor - shapes	0.0004	0.0007	11960.01	-0.0009	0.0017	0.58	0.56289
motor - story	0.0018	0.0007	11969.74	0.0005	0.0031	2.64	0.00836
motor - ToM	0.0015	0.0007	11965.26	0.0001	0.0028	2.18	0.02946
punish - random	0.0009	0.0007	11955.97	-0.0004	0.0022	1.33	0.18207
punish - relational	-0.0003	0.0007	11959.18	-0.0016	0.0010	-0.49	0.62727
punish - reward	-0.0005	0.0007	11916.92	-0.0018	0.0008	-0.77	0.44202
punish - shapes	-0.0007	0.0007	11951.19	-0.0020	0.0006	-1.11	0.26757
punish - story	0.0007	0.0007	11971.87	-0.0007	0.0020	1.00	0.31861
punish - ToM	0.0003	0.0007	11955.97	-0.0010	0.0016	0.50	0.61652
random - relational	-0.0012	0.0007	11939.45	-0.0025	0.0001	-1.80	0.07139
random - reward	-0.0014	0.0007	11955.97	-0.0027	-0.0001	-2.10	0.03585
random - shapes	-0.0016	0.0007	11931.81	-0.0029	-0.0003	-2.43	0.01493
random - story	-0.0002	0.0007	11947.55	-0.0015	0.0011	-0.31	0.75782
random - ToM	-0.0006	0.0007	11916.92	-0.0019	0.0008	-0.83	0.40539
relational - reward	-0.0002	0.0007	11959.18	-0.0015	0.0011	-0.27	0.78576
relational - shapes	-0.0004	0.0007	11931.56	-0.0017	0.0009	-0.61	0.54029
relational - story	0.0010	0.0007	11940.00	-0.0003	0.0023	1.46	0.14377
relational - ToM	0.0007	0.0007	11939.45	-0.0007	0.0020	0.98	0.32748
reward - shapes	-0.0002	0.0007	11951.19	-0.0015	0.0011	-0.35	0.72967
reward - story	0.0012	0.0007	11971.87	-0.0001	0.0025	1.75	0.08089
reward - ToM	0.0008	0.0007	11955.97	-0.0005	0.0021	1.27	0.20583
shapes - story	0.0014	0.0007	11950.03	0.0001	0.0028	2.08	0.03793
shapes - ToM	0.0011	0.0007	11931.81	-0.0002	0.0024	1.60	0.10866
story - ToM	-0.0003	0.0007	11947.55	-0.0017	0.0010	-0.51	0.61295

Supplementary Table 7.5 Agreeableness* task condition interactions along D1 (primary-association)

contrast	β	SE	df	CL lower	CL upper	t.ratio	p.value
0back - 2back	0.0004	0.0007	11916.92	-0.0009	0.0017	0.56	0.57366
0back - faces	0.0009	0.0007	11948.10	-0.0004	0.0023	1.39	0.16415
0back - match	0.0012	0.0007	11953.49	-0.0001	0.0025	1.78	0.07558
0back - math	-0.0007	0.0007	11978.23	-0.0020	0.0007	-0.98	0.32575
0back - motor	0.0003	0.0007	11930.91	-0.0010	0.0016	0.44	0.66224
0back - punish	0.0006	0.0007	11929.93	-0.0007	0.0019	0.88	0.37864
0back - random	-0.0008	0.0007	11960.92	-0.0022	0.0005	-1.26	0.20636
0back - relational	0.0012	0.0007	11953.49	-0.0001	0.0026	1.82	0.06929
0back - reward	0.0002	0.0007	11929.93	-0.0011	0.0015	0.32	0.75102
0back - shapes	0.0011	0.0007	11948.10	-0.0002	0.0025	1.71	0.08771
0back - story	-0.0007	0.0007	11978.23	-0.0021	0.0006	-1.04	0.29816
0back - ToM	0.0000	0.0007	11960.92	-0.0013	0.0013	0.03	0.97449
2back - faces	0.0006	0.0007	11948.10	-0.0008	0.0019	0.83	0.40494
2back - match	0.0008	0.0007	11953.49	-0.0005	0.0022	1.22	0.22194
2back - math	-0.0011	0.0007	11978.23	-0.0024	0.0003	-1.52	0.12732
2back - motor	-0.0001	0.0007	11930.91	-0.0014	0.0012	-0.12	0.90276
2back - punish	0.0002	0.0007	11929.93	-0.0011	0.0015	0.32	0.75020
2back - random	-0.0012	0.0007	11960.92	-0.0025	0.0001	-1.82	0.06818
2back - relational	0.0009	0.0007	11953.49	-0.0005	0.0022	1.26	0.20731
2back - reward	-0.0002	0.0007	11929.93	-0.0015	0.0011	-0.24	0.80665
2back - shapes	0.0008	0.0007	11948.10	-0.0005	0.0021	1.15	0.25048
2back - story	-0.0011	0.0007	11978.23	-0.0025	0.0003	-1.58	0.11355
2back - ToM	-0.0004	0.0007	11960.92	-0.0017	0.0010	-0.53	0.59732
faces - match	0.0003	0.0007	11928.40	-0.0011	0.0016	0.39	0.69574
faces - math	-0.0016	0.0007	11957.50	-0.0030	-0.0003	-2.32	0.02021
faces - motor	-0.0006	0.0007	11954.54	-0.0020	0.0007	-0.95	0.34255
faces - punish	-0.0003	0.0007	11945.55	-0.0017	0.0010	-0.52	0.60560
faces - random	-0.0018	0.0007	11941.96	-0.0031	-0.0005	-2.65	0.00815
faces - relational	0.0003	0.0007	11928.40	-0.0010	0.0016	0.43	0.66681
faces - reward	-0.0007	0.0007	11945.55	-0.0020	0.0006	-1.08	0.28205
faces - shapes	0.0002	0.0007	11916.92	-0.0011	0.0015	0.32	0.75272
faces - story	-0.0017	0.0007	11957.50	-0.0030	-0.0003	-2.38	0.01732
faces - ToM	-0.0009	0.0007	11941.96	-0.0022	0.0004	-1.36	0.17479
match - math	-0.0019	0.0007	11953.39	-0.0033	-0.0005	-2.69	0.00710
match - motor	-0.0009	0.0007	11949.67	-0.0022	0.0004	-1.34	0.18170
match - punish	-0.0006	0.0007	11951.47	-0.0019	0.0007	-0.91	0.36476
match - random	-0.0020	0.0007	11950.08	-0.0034	-0.0007	-3.02	0.00250

match - relational	0.0000	0.0007	11916.92	-0.0013	0.0014	0.04	0.96866
match - reward	-0.0010	0.0007	11951.47	-0.0023	0.0003	-1.46	0.14354
match - shapes	-0.0001	0.0007	11928.40	-0.0014	0.0013	-0.08	0.93804
match - story	-0.0019	0.0007	11953.39	-0.0033	-0.0006	-2.75	0.00597
match - ToM	-0.0012	0.0007	11950.08	-0.0025	0.0001	-1.74	0.08156
math - motor	0.0010	0.0007	11975.70	-0.0004	0.0023	1.40	0.16182
math - punish	0.0013	0.0007	11973.24	-0.0001	0.0026	1.83	0.06703
math - random	-0.0002	0.0007	11965.38	-0.0015	0.0012	-0.24	0.81059
math - relational	0.0019	0.0007	11953.39	0.0005	0.0033	2.73	0.00632
math - reward	0.0009	0.0007	11973.24	-0.0005	0.0023	1.29	0.19756
math - shapes	0.0018	0.0007	11957.50	0.0005	0.0032	2.63	0.00859
math - story	0.0000	0.0007	11916.92	-0.0014	0.0014	-0.06	0.95526
math - ToM	0.0007	0.0007	11965.38	-0.0007	0.0021	1.01	0.31141
motor - punish	0.0003	0.0007	11933.07	-0.0010	0.0016	0.44	0.66095
motor - random	-0.0011	0.0007	11970.77	-0.0025	0.0002	-1.69	0.09090
motor - relational	0.0009	0.0007	11949.67	-0.0004	0.0023	1.38	0.16914
motor - reward	-0.0001	0.0007	11933.07	-0.0014	0.0012	-0.12	0.90354
motor - shapes	0.0009	0.0007	11954.54	-0.0005	0.0022	1.26	0.20638
motor - story	-0.0010	0.0007	11975.70	-0.0024	0.0004	-1.46	0.14529
motor - ToM	-0.0003	0.0007	11970.77	-0.0016	0.0011	-0.40	0.68676
punish - random	-0.0014	0.0007	11959.73	-0.0028	-0.0001	-2.14	0.03233
punish - relational	0.0006	0.0007	11951.47	-0.0007	0.0020	0.95	0.34418
punish - reward	-0.0004	0.0007	11916.92	-0.0017	0.0009	-0.56	0.57325
punish - shapes	0.0006	0.0007	11945.55	-0.0008	0.0019	0.83	0.40504
punish - story	-0.0013	0.0007	11973.24	-0.0027	0.0000	-1.89	0.05887
punish - ToM	-0.0006	0.0007	11959.73	-0.0019	0.0007	-0.85	0.39795
random - relational	0.0021	0.0007	11950.08	0.0007	0.0034	3.06	0.00219
random - reward	0.0011	0.0007	11959.73	-0.0003	0.0024	1.58	0.11427
random - shapes	0.0020	0.0007	11941.96	0.0007	0.0033	2.96	0.00306
random - story	0.0001	0.0007	11965.38	-0.0012	0.0015	0.18	0.85553
random - ToM	0.0009	0.0007	11916.92	-0.0004	0.0022	1.30	0.19527
relational - reward	-0.0010	0.0007	11951.47	-0.0023	0.0003	-1.50	0.13301
relational - shapes	-0.0001	0.0007	11928.40	-0.0014	0.0013	-0.12	0.90672
relational - story	-0.0020	0.0007	11953.39	-0.0033	-0.0006	-2.79	0.00531
relational - ToM	-0.0012	0.0007	11950.08	-0.0025	0.0001	-1.78	0.07487
reward - shapes	0.0009	0.0007	11945.55	-0.0004	0.0023	1.39	0.16392
reward - story	-0.0009	0.0007	11973.24	-0.0023	0.0004	-1.35	0.17824
reward - ToM	-0.0002	0.0007	11959.73	-0.0015	0.0011	-0.28	0.77627
shapes - story	-0.0019	0.0007	11957.50	-0.0032	-0.0005	-2.69	0.00724
shapes - ToM	-0.0011	0.0007	11941.96	-0.0024	0.0002	-1.67	0.09437
story - ToM	0.0007	0.0007	11965.38	-0.0006	0.0021	1.07	0.28468

Supplementary Table 8.1 Neuroticism* task condition interactions along D2 (motor- visual)

contrast	β	SE	df	CL_{lower}	CL_{upper}	t.ratio	p.value
0back - 2back	0.0009	0.0004	11922.26	0.0001	0.0017	2.22	0.02632
0back - faces	0.0003	0.0004	11971.88	-0.0005	0.0012	0.82	0.41306
0back - match	0.0000	0.0004	11975.31	-0.0008	0.0008	-0.03	0.97897
0back - math	-0.0006	0.0004	11983.92	-0.0015	0.0002	-1.47	0.14239
0back - motor	-0.0010	0.0004	11940.41	-0.0018	-0.0002	-2.34	0.01921
0back - punish	-0.0001	0.0004	11943.31	-0.0009	0.0007	-0.15	0.87940
0back - random	-0.0004	0.0004	11978.05	-0.0012	0.0004	-0.97	0.33209
0back - relational	0.0001	0.0004	11975.31	-0.0007	0.0010	0.33	0.73810
0back - reward	-0.0001	0.0004	11943.31	-0.0009	0.0007	-0.17	0.86735
0back - shapes	0.0001	0.0004	11971.88	-0.0007	0.0009	0.19	0.85175
0back - story	-0.0008	0.0004	11983.92	-0.0016	0.0000	-1.85	0.06467
0back - ToM	-0.0002	0.0004	11978.05	-0.0010	0.0006	-0.55	0.57908
2back - faces	-0.0006	0.0004	11971.89	-0.0014	0.0002	-1.38	0.16781
2back - match	-0.0009	0.0004	11975.32	-0.0018	-0.0001	-2.21	0.02690
2back - math	-0.0015	0.0004	11983.93	-0.0024	-0.0007	-3.62	0.00030
2back - motor	-0.0019	0.0004	11940.42	-0.0027	-0.0011	-4.55	0.00001
2back - punish	-0.0010	0.0004	11943.32	-0.0018	-0.0002	-2.37	0.01772
2back - random	-0.0013	0.0004	11978.06	-0.0021	-0.0005	-3.17	0.00151
2back - relational	-0.0008	0.0004	11975.32	-0.0016	0.0000	-1.85	0.06398
2back - reward	-0.0010	0.0004	11943.32	-0.0018	-0.0002	-2.39	0.01700
2back - shapes	-0.0008	0.0004	11971.89	-0.0017	0.0000	-2.01	0.04435
2back - story	-0.0017	0.0004	11983.93	-0.0025	-0.0009	-4.00	0.00006
2back - ToM	-0.0011	0.0004	11978.06	-0.0020	-0.0003	-2.76	0.00581
faces - match	-0.0004	0.0004	11934.81	-0.0012	0.0005	-0.84	0.40362
faces - math	-0.0010	0.0004	11955.45	-0.0018	-0.0001	-2.25	0.02429
faces - motor	-0.0013	0.0004	11979.99	-0.0021	-0.0005	-3.13	0.00174
faces - punish	-0.0004	0.0004	11970.40	-0.0012	0.0004	-0.97	0.33256
faces - random	-0.0007	0.0004	11938.33	-0.0016	0.0001	-1.78	0.07534
faces - relational	-0.0002	0.0004	11934.81	-0.0010	0.0006	-0.48	0.63347
faces - reward	-0.0004	0.0004	11970.40	-0.0012	0.0004	-0.98	0.32506
faces - shapes	-0.0003	0.0004	11922.26	-0.0011	0.0006	-0.63	0.53021
faces - story	-0.0011	0.0004	11955.45	-0.0020	-0.0003	-2.63	0.00853
faces - ToM	-0.0006	0.0004	11938.33	-0.0014	0.0002	-1.37	0.17191
match - math	-0.0006	0.0004	11949.32	-0.0015	0.0002	-1.42	0.15425
match - motor	-0.0010	0.0004	11976.97	-0.0018	-0.0001	-2.28	0.02258
match - punish	-0.0001	0.0004	11978.71	-0.0009	0.0008	-0.12	0.90202
match - random	-0.0004	0.0004	11945.62	-0.0012	0.0004	-0.93	0.35134
match - relational	0.0002	0.0004	11922.26	-0.0007	0.0010	0.36	0.72119
match - reward	-0.0001	0.0004	11978.71	-0.0009	0.0008	-0.14	0.89011

match - shapes	0.0001	0.0004	11934.81	-0.0007	0.0009	0.21	0.83297
match - story	-0.0008	0.0004	11949.32	-0.0016	0.0001	-1.80	0.07170
match - ToM	-0.0002	0.0004	11945.62	-0.0010	0.0006	-0.52	0.60181
math - motor	-0.0003	0.0004	11988.96	-0.0012	0.0005	-0.81	0.41796
math - punish	0.0006	0.0004	11991.05	-0.0003	0.0014	1.32	0.18700
math - random	0.0002	0.0004	11960.60	-0.0006	0.0011	0.52	0.60483
math - relational	0.0008	0.0004	11949.32	-0.0001	0.0016	1.78	0.07578
math - reward	0.0006	0.0004	11991.05	-0.0003	0.0014	1.30	0.19200
math - shapes	0.0007	0.0004	11955.45	-0.0001	0.0015	1.64	0.10134
math - story	-0.0002	0.0004	11922.26	-0.0010	0.0007	-0.37	0.71011
math - ToM	0.0004	0.0004	11960.60	-0.0004	0.0012	0.92	0.35696
motor - punish	0.0009	0.0004	11943.13	0.0001	0.0017	2.19	0.02850
motor - random	0.0006	0.0004	11987.87	-0.0003	0.0014	1.36	0.17427
motor - relational	0.0011	0.0004	11976.97	0.0003	0.0019	2.64	0.00830
motor - reward	0.0009	0.0004	11943.13	0.0001	0.0017	2.18	0.02962
motor - shapes	0.0011	0.0004	11979.99	0.0002	0.0019	2.50	0.01230
motor - story	0.0002	0.0004	11988.96	-0.0007	0.0010	0.43	0.66654
motor - ToM	0.0007	0.0004	11987.87	-0.0001	0.0016	1.77	0.07643
punish - random	-0.0003	0.0004	11976.70	-0.0012	0.0005	-0.82	0.41256
punish - relational	0.0002	0.0004	11978.71	-0.0006	0.0010	0.48	0.62851
punish - reward	0.0000	0.0004	11922.26	-0.0008	0.0008	-0.02	0.98779
punish - shapes	0.0001	0.0004	11970.40	-0.0007	0.0010	0.34	0.73597
punish - story	-0.0007	0.0004	11991.05	-0.0016	0.0001	-1.70	0.08912
punish - ToM	-0.0002	0.0004	11976.70	-0.0010	0.0006	-0.40	0.68610
random - relational	0.0005	0.0004	11945.62	-0.0003	0.0014	1.29	0.19665
random - reward	0.0003	0.0004	11976.70	-0.0005	0.0012	0.80	0.42127
random - shapes	0.0005	0.0004	11938.33	-0.0003	0.0013	1.15	0.25040
random - story	-0.0004	0.0004	11960.60	-0.0012	0.0005	-0.90	0.37000
random - ToM	0.0002	0.0004	11922.26	-0.0006	0.0010	0.41	0.67903
relational - reward	-0.0002	0.0004	11978.71	-0.0010	0.0006	-0.50	0.61786
relational - shapes	-0.0001	0.0004	11934.81	-0.0009	0.0008	-0.15	0.88280
relational - story	-0.0009	0.0004	11949.32	-0.0018	-0.0001	-2.15	0.03140
relational - ToM	-0.0004	0.0004	11945.62	-0.0012	0.0005	-0.88	0.37833
reward - shapes	0.0001	0.0004	11970.40	-0.0007	0.0010	0.35	0.72458
reward - story	-0.0007	0.0004	11991.05	-0.0016	0.0001	-1.69	0.09195
reward - ToM	-0.0002	0.0004	11976.70	-0.0010	0.0007	-0.39	0.69730
shapes - story	-0.0009	0.0004	11955.45	-0.0017	0.0000	-2.02	0.04377
shapes - ToM	-0.0003	0.0004	11938.33	-0.0011	0.0005	-0.74	0.46109
story - ToM	0.0006	0.0004	11960.60	-0.0003	0.0014	1.30	0.19355

Supplementary Table 8.2 Openness* task condition interactions along D2 (motor- visual)

contrast	β	SE	df	CL lower	CL upper	t.ratio	p.value
0back - 2back	-0.0005	0.0004	11922.26	-0.0014	0.0003	-1.20	0.23013
0back - faces	-0.0005	0.0004	11968.02	-0.0014	0.0004	-1.11	0.26817
0back - match	0.0006	0.0004	11972.91	-0.0003	0.0014	1.24	0.21323
0back - math	-0.0011	0.0005	11987.09	-0.0019	-0.0002	-2.32	0.02041
0back - motor	0.0000	0.0004	11942.86	-0.0009	0.0008	-0.09	0.92739
0back - punish	0.0006	0.0004	11940.58	-0.0003	0.0014	1.35	0.17768
0back - random	-0.0006	0.0004	11969.08	-0.0014	0.0003	-1.28	0.19983
0back - relational	0.0000	0.0004	11972.91	-0.0009	0.0008	-0.11	0.91392
0back - reward	0.0004	0.0004	11940.58	-0.0005	0.0012	0.88	0.38049
0back - shapes	0.0003	0.0004	11968.02	-0.0006	0.0012	0.69	0.49277
0back - story	-0.0006	0.0005	11987.09	-0.0015	0.0003	-1.35	0.17814
0back - ToM	-0.0001	0.0004	11969.08	-0.0009	0.0008	-0.12	0.90412
2back - faces	0.0000	0.0004	11968.02	-0.0008	0.0009	0.08	0.93651
2back - match	0.0011	0.0004	11972.92	0.0002	0.0020	2.43	0.01532
2back - math	-0.0005	0.0005	11987.09	-0.0014	0.0004	-1.16	0.24605
2back - motor	0.0005	0.0004	11942.87	-0.0004	0.0013	1.10	0.27091
2back - punish	0.0011	0.0004	11940.59	0.0003	0.0020	2.55	0.01077
2back - random	0.0000	0.0004	11969.08	-0.0009	0.0008	-0.09	0.92713
2back - relational	0.0005	0.0004	11972.92	-0.0004	0.0014	1.07	0.28364
2back - reward	0.0009	0.0004	11940.59	0.0001	0.0018	2.08	0.03758
2back - shapes	0.0008	0.0004	11968.02	0.0000	0.0017	1.87	0.06110
2back - story	-0.0001	0.0005	11987.09	-0.0010	0.0008	-0.19	0.85120
2back - ToM	0.0005	0.0004	11969.08	-0.0004	0.0013	1.07	0.28456
faces - match	0.0010	0.0004	11942.02	0.0002	0.0019	2.33	0.01987
faces - math	-0.0006	0.0005	11963.12	-0.0015	0.0003	-1.23	0.21911
faces - motor	0.0005	0.0004	11978.21	-0.0004	0.0013	1.01	0.31222
faces - punish	0.0011	0.0004	11968.99	0.0002	0.0019	2.45	0.01449
faces - random	-0.0001	0.0004	11933.03	-0.0009	0.0008	-0.17	0.86490
faces - relational	0.0004	0.0004	11942.02	-0.0004	0.0013	0.99	0.32419
faces - reward	0.0009	0.0004	11968.99	0.0000	0.0017	1.98	0.04785
faces - shapes	0.0008	0.0004	11922.26	-0.0001	0.0017	1.78	0.07483
faces - story	-0.0001	0.0005	11963.12	-0.0010	0.0008	-0.26	0.79220
faces - ToM	0.0004	0.0004	11933.03	-0.0004	0.0013	0.98	0.32550
match - math	-0.0016	0.0005	11952.08	-0.0025	-0.0007	-3.50	0.00047
match - motor	-0.0006	0.0004	11975.19	-0.0015	0.0003	-1.33	0.18428
match - punish	0.0000	0.0004	11980.90	-0.0008	0.0009	0.08	0.93746
match - random	-0.0011	0.0004	11946.66	-0.0020	-0.0002	-2.50	0.01227
match - relational	-0.0006	0.0005	11922.26	-0.0015	0.0003	-1.34	0.18106
match - reward	-0.0002	0.0004	11980.90	-0.0010	0.0007	-0.39	0.70024

match - shapes	-0.0003	0.0004	11942.02	-0.0011	0.0006	-0.56	0.57638
match - story	-0.0012	0.0005	11952.08	-0.0021	-0.0003	-2.54	0.01113
match - ToM	-0.0006	0.0004	11946.66	-0.0015	0.0003	-1.36	0.17444
math - motor	0.0010	0.0005	11986.21	0.0001	0.0019	2.22	0.02650
math - punish	0.0016	0.0005	11993.86	0.0008	0.0025	3.63	0.00029
math - random	0.0005	0.0005	11961.42	-0.0004	0.0014	1.07	0.28622
math - relational	0.0010	0.0005	11952.08	0.0001	0.0019	2.19	0.02875
math - reward	0.0014	0.0005	11993.86	0.0005	0.0023	3.17	0.00152
math - shapes	0.0014	0.0005	11963.12	0.0005	0.0023	2.97	0.00301
math - story	0.0004	0.0005	11922.26	-0.0005	0.0014	0.95	0.34337
math - ToM	0.0010	0.0005	11961.42	0.0001	0.0019	2.19	0.02836
motor - punish	0.0006	0.0004	11943.46	-0.0002	0.0015	1.43	0.15229
motor - random	-0.0005	0.0004	11977.18	-0.0014	0.0003	-1.18	0.23640
motor - relational	0.0000	0.0004	11975.19	-0.0009	0.0009	-0.02	0.98582
motor - reward	0.0004	0.0004	11943.46	-0.0004	0.0013	0.96	0.33541
motor - shapes	0.0003	0.0004	11978.21	-0.0005	0.0012	0.77	0.44008
motor - story	-0.0006	0.0005	11986.21	-0.0015	0.0003	-1.25	0.21074
motor - ToM	0.0000	0.0004	11977.18	-0.0009	0.0009	-0.03	0.97665
punish - random	-0.0012	0.0004	11964.15	-0.0020	-0.0003	-2.63	0.00868
punish - relational	-0.0006	0.0004	11980.90	-0.0015	0.0002	-1.43	0.15135
punish - reward	-0.0002	0.0004	11922.26	-0.0011	0.0006	-0.47	0.63639
punish - shapes	-0.0003	0.0004	11968.99	-0.0012	0.0006	-0.65	0.51794
punish - story	-0.0012	0.0005	11993.86	-0.0021	-0.0003	-2.65	0.00800
punish - ToM	-0.0006	0.0004	11964.15	-0.0015	0.0002	-1.46	0.14440
random - relational	0.0005	0.0004	11946.66	-0.0004	0.0014	1.16	0.24700
random - reward	0.0009	0.0004	11964.15	0.0001	0.0018	2.16	0.03101
random - shapes	0.0009	0.0004	11933.03	0.0000	0.0017	1.96	0.05047
random - story	0.0000	0.0005	11961.42	-0.0009	0.0008	-0.10	0.92181
random - ToM	0.0005	0.0004	11922.26	-0.0004	0.0014	1.16	0.24712
relational - reward	0.0004	0.0004	11980.90	-0.0004	0.0013	0.97	0.33136
relational - shapes	0.0004	0.0004	11942.02	-0.0005	0.0012	0.78	0.43275
relational - story	-0.0006	0.0005	11952.08	-0.0015	0.0003	-1.23	0.22023
relational - ToM	0.0000	0.0004	11946.66	-0.0009	0.0009	-0.01	0.99101
reward - shapes	-0.0001	0.0004	11968.99	-0.0009	0.0008	-0.18	0.85695
reward - story	-0.0010	0.0005	11993.86	-0.0019	-0.0001	-2.20	0.02802
reward - ToM	-0.0004	0.0004	11964.15	-0.0013	0.0004	-0.99	0.32126
shapes - story	-0.0009	0.0005	11963.12	-0.0018	0.0000	-2.00	0.04532
shapes - ToM	-0.0004	0.0004	11933.03	-0.0012	0.0005	-0.80	0.42214
story - ToM	0.0006	0.0005	11961.42	-0.0003	0.0015	1.22	0.22093

Supplementary Table 8.3 Conscientiousness* task condition interactions along D2 (motor-visual)

contrast	β	SE	df	CL lower	CL upper	t.ratio	p.value
0back - 2back	0.0006	0.0005	11922.26	-0.0004	0.0015	1.13	0.25828
0back - faces	0.0002	0.0005	11980.41	-0.0007	0.0012	0.49	0.62476
0back - match	-0.0007	0.0005	11984.50	-0.0017	0.0003	-1.35	0.17580
0back - math	-0.0001	0.0005	12000.76	-0.0011	0.0009	-0.16	0.87072
0back - motor	-0.0004	0.0005	11936.32	-0.0014	0.0006	-0.78	0.43314
0back - punish	-0.0002	0.0005	11943.83	-0.0012	0.0007	-0.46	0.64431
0back - random	-0.0008	0.0005	11987.75	-0.0018	0.0002	-1.59	0.11190
0back - relational	-0.0003	0.0005	11984.50	-0.0013	0.0007	-0.52	0.60520
0back - reward	-0.0002	0.0005	11943.83	-0.0012	0.0008	-0.44	0.65651
0back - shapes	0.0000	0.0005	11980.41	-0.0010	0.0010	0.01	0.99086
0back - story	-0.0007	0.0005	12000.76	-0.0018	0.0003	-1.44	0.14909
0back - ToM	-0.0005	0.0005	11987.75	-0.0015	0.0005	-0.90	0.36782
2back - faces	-0.0003	0.0005	11980.42	-0.0013	0.0007	-0.63	0.52922
2back - match	-0.0013	0.0005	11984.51	-0.0023	-0.0003	-2.47	0.01369
2back - math	-0.0007	0.0005	12000.77	-0.0017	0.0004	-1.26	0.20890
2back - motor	-0.0010	0.0005	11936.32	-0.0020	0.0000	-1.91	0.05647
2back - punish	-0.0008	0.0005	11943.84	-0.0018	0.0002	-1.59	0.11083
2back - random	-0.0014	0.0005	11987.76	-0.0024	-0.0004	-2.71	0.00678
2back - relational	-0.0008	0.0005	11984.51	-0.0018	0.0002	-1.63	0.10337
2back - reward	-0.0008	0.0005	11943.84	-0.0018	0.0002	-1.58	0.11468
2back - shapes	-0.0006	0.0005	11980.42	-0.0016	0.0004	-1.11	0.26836
2back - story	-0.0013	0.0005	12000.77	-0.0023	-0.0003	-2.54	0.01120
2back - ToM	-0.0010	0.0005	11987.76	-0.0020	0.0000	-2.02	0.04356
faces - match	-0.0009	0.0005	11934.36	-0.0019	0.0001	-1.83	0.06734
faces - math	-0.0003	0.0005	11957.43	-0.0014	0.0007	-0.64	0.52385
faces - motor	-0.0006	0.0005	11986.40	-0.0016	0.0004	-1.26	0.20686
faces - punish	-0.0005	0.0005	11975.53	-0.0015	0.0005	-0.95	0.34314
faces - random	-0.0011	0.0005	11939.47	-0.0021	-0.0001	-2.07	0.03881
faces - relational	-0.0005	0.0005	11934.36	-0.0015	0.0005	-1.00	0.31856
faces - reward	-0.0005	0.0005	11975.53	-0.0015	0.0005	-0.93	0.35175
faces - shapes	-0.0002	0.0005	11922.26	-0.0012	0.0008	-0.48	0.63477
faces - story	-0.0010	0.0005	11957.43	-0.0020	0.0000	-1.91	0.05615
faces - ToM	-0.0007	0.0005	11939.47	-0.0017	0.0003	-1.38	0.16723
match - math	0.0006	0.0005	11955.54	-0.0004	0.0016	1.16	0.24752
match - motor	0.0003	0.0005	11983.22	-0.0007	0.0013	0.58	0.56508
match - punish	0.0005	0.0005	11982.11	-0.0005	0.0015	0.90	0.36626
match - random	-0.0001	0.0005	11948.16	-0.0011	0.0009	-0.23	0.82127

match - relational	0.0004	0.0005	11922.26	-0.0006	0.0014	0.83	0.40759
match - reward	0.0005	0.0005	11982.11	-0.0005	0.0015	0.92	0.35748
match - shapes	0.0007	0.0005	11934.36	-0.0003	0.0017	1.36	0.17469
match - story	-0.0001	0.0005	11955.54	-0.0011	0.0010	-0.11	0.91265
match - ToM	0.0002	0.0005	11948.16	-0.0008	0.0012	0.46	0.64899
math - motor	-0.0003	0.0005	11992.67	-0.0013	0.0007	-0.60	0.55006
math - punish	-0.0001	0.0005	11997.02	-0.0012	0.0009	-0.28	0.77646
math - random	-0.0007	0.0005	11966.26	-0.0017	0.0003	-1.38	0.16635
math - relational	-0.0002	0.0005	11955.54	-0.0012	0.0008	-0.34	0.73232
math - reward	-0.0001	0.0005	11997.02	-0.0012	0.0009	-0.27	0.78907
math - shapes	0.0001	0.0005	11957.43	-0.0009	0.0011	0.17	0.86270
math - story	-0.0007	0.0005	11922.26	-0.0017	0.0004	-1.25	0.21112
math - ToM	-0.0004	0.0005	11966.26	-0.0014	0.0006	-0.71	0.47527
motor - punish	0.0002	0.0005	11949.01	-0.0008	0.0012	0.33	0.74387
motor - random	-0.0004	0.0005	11993.60	-0.0014	0.0006	-0.81	0.42050
motor - relational	0.0001	0.0005	11983.22	-0.0009	0.0011	0.26	0.79689
motor - reward	0.0002	0.0005	11949.01	-0.0008	0.0012	0.34	0.73116
motor - shapes	0.0004	0.0005	11986.40	-0.0006	0.0014	0.79	0.43115
motor - story	-0.0004	0.0005	11992.67	-0.0014	0.0007	-0.68	0.49854
motor - ToM	-0.0001	0.0005	11993.60	-0.0011	0.0009	-0.12	0.90440
punish - random	-0.0006	0.0005	11976.28	-0.0016	0.0004	-1.14	0.25506
punish - relational	0.0000	0.0005	11982.11	-0.0010	0.0010	-0.06	0.94897
punish - reward	0.0000	0.0005	11922.26	-0.0010	0.0010	0.02	0.98643
punish - shapes	0.0002	0.0005	11975.53	-0.0008	0.0012	0.47	0.63925
punish - story	-0.0005	0.0005	11997.02	-0.0015	0.0005	-1.00	0.31726
punish - ToM	-0.0002	0.0005	11976.28	-0.0012	0.0008	-0.45	0.65521
random - relational	0.0005	0.0005	11948.16	-0.0005	0.0015	1.06	0.29033
random - reward	0.0006	0.0005	11976.28	-0.0004	0.0016	1.15	0.24812
random - shapes	0.0008	0.0005	11939.47	-0.0002	0.0018	1.59	0.11147
random - story	0.0001	0.0005	11966.26	-0.0010	0.0011	0.11	0.91076
random - ToM	0.0003	0.0005	11922.26	-0.0006	0.0013	0.69	0.49294
relational - reward	0.0000	0.0005	11982.11	-0.0010	0.0010	0.08	0.93570
relational - shapes	0.0003	0.0005	11934.36	-0.0007	0.0013	0.53	0.59940
relational - story	-0.0005	0.0005	11955.54	-0.0015	0.0005	-0.92	0.35546
relational - ToM	-0.0002	0.0005	11948.16	-0.0012	0.0008	-0.38	0.70666
reward - shapes	0.0002	0.0005	11975.53	-0.0008	0.0012	0.45	0.65129
reward - story	-0.0005	0.0005	11997.02	-0.0015	0.0005	-1.02	0.30939
reward - ToM	-0.0002	0.0005	11976.28	-0.0012	0.0008	-0.46	0.64314
shapes - story	-0.0008	0.0005	11957.43	-0.0018	0.0003	-1.45	0.14833
shapes - ToM	-0.0005	0.0005	11939.47	-0.0015	0.0005	-0.91	0.36464
story - ToM	0.0003	0.0005	11966.26	-0.0007	0.0013	0.56	0.57680

Supplementary Table 8.4 Extraversion* task condition interactions along D2 (motor- visual)

contrast	β	SE	df	CL lower	CL upper	t.ratio	p.value
0back - 2back	0.0000	0.0005	11922.26	-0.0009	0.0010	0.07	0.94046
0back - faces	-0.0003	0.0005	11962.48	-0.0013	0.0006	-0.67	0.50264
0back - match	-0.0002	0.0005	11971.66	-0.0012	0.0008	-0.41	0.68107
0back - math	-0.0010	0.0005	11985.18	-0.0019	0.0000	-1.92	0.05503
0back - motor	-0.0014	0.0005	11943.04	-0.0024	-0.0005	-2.91	0.00359
0back - punish	-0.0005	0.0005	11933.78	-0.0015	0.0004	-1.11	0.26782
0back - random	0.0002	0.0005	11964.27	-0.0007	0.0012	0.49	0.62455
0back - relational	-0.0004	0.0005	11971.66	-0.0014	0.0006	-0.80	0.42657
0back - reward	-0.0009	0.0005	11933.78	-0.0019	0.0000	-1.93	0.05401
0back - shapes	-0.0008	0.0005	11962.48	-0.0018	0.0002	-1.64	0.10030
0back - story	-0.0010	0.0005	11985.18	-0.0020	0.0000	-2.00	0.04560
0back - ToM	0.0001	0.0005	11964.27	-0.0009	0.0010	0.18	0.85626
2back - faces	-0.0004	0.0005	11962.48	-0.0013	0.0006	-0.74	0.45654
2back - match	-0.0002	0.0005	11971.66	-0.0012	0.0007	-0.48	0.62792
2back - math	-0.0010	0.0005	11985.18	-0.0020	0.0000	-1.99	0.04644
2back - motor	-0.0015	0.0005	11943.05	-0.0024	-0.0005	-2.99	0.00282
2back - punish	-0.0006	0.0005	11933.79	-0.0015	0.0004	-1.18	0.23688
2back - random	0.0002	0.0005	11964.27	-0.0008	0.0012	0.42	0.67809
2back - relational	-0.0004	0.0005	11971.66	-0.0014	0.0005	-0.87	0.38499
2back - reward	-0.0010	0.0005	11933.79	-0.0019	0.0000	-2.00	0.04534
2back - shapes	-0.0008	0.0005	11962.48	-0.0018	0.0001	-1.72	0.08587
2back - story	-0.0010	0.0005	11985.18	-0.0020	-0.0001	-2.07	0.03828
2back - ToM	0.0001	0.0005	11964.27	-0.0009	0.0010	0.11	0.91496
faces - match	0.0001	0.0005	11938.46	-0.0008	0.0011	0.25	0.79976
faces - math	-0.0006	0.0005	11958.76	-0.0016	0.0004	-1.26	0.20857
faces - motor	-0.0011	0.0005	11969.31	-0.0021	-0.0001	-2.23	0.02568
faces - punish	-0.0002	0.0005	11959.56	-0.0012	0.0007	-0.43	0.66665
faces - random	0.0006	0.0005	11938.04	-0.0004	0.0015	1.16	0.24720
faces - relational	-0.0001	0.0005	11938.46	-0.0010	0.0009	-0.13	0.89705
faces - reward	-0.0006	0.0005	11959.56	-0.0016	0.0004	-1.25	0.21315
faces - shapes	-0.0005	0.0005	11922.26	-0.0014	0.0005	-0.97	0.33169
faces - story	-0.0007	0.0005	11958.76	-0.0017	0.0003	-1.34	0.18097
faces - ToM	0.0004	0.0005	11938.04	-0.0005	0.0014	0.85	0.39537
match - math	-0.0008	0.0005	11947.83	-0.0017	0.0002	-1.50	0.13391
match - motor	-0.0012	0.0005	11963.58	-0.0022	-0.0003	-2.47	0.01354
match - punish	-0.0003	0.0005	11968.54	-0.0013	0.0006	-0.68	0.49511
match - random	0.0004	0.0005	11946.69	-0.0005	0.0014	0.89	0.37099
match - relational	-0.0002	0.0005	11922.26	-0.0012	0.0008	-0.38	0.70337
match - reward	-0.0007	0.0005	11968.54	-0.0017	0.0002	-1.49	0.13615

match - shapes	-0.0006	0.0005	11938.46	-0.0016	0.0004	-1.22	0.22366
match - story	-0.0008	0.0005	11947.83	-0.0018	0.0002	-1.58	0.11442
match - ToM	0.0003	0.0005	11946.69	-0.0007	0.0013	0.59	0.55544
math - motor	-0.0005	0.0005	11979.98	-0.0015	0.0005	-0.94	0.34917
math - punish	0.0004	0.0005	11982.45	-0.0006	0.0014	0.84	0.40061
math - random	0.0012	0.0005	11955.72	0.0002	0.0022	2.40	0.01663
math - relational	0.0006	0.0005	11947.83	-0.0004	0.0016	1.12	0.26151
math - reward	0.0000	0.0005	11982.45	-0.0010	0.0010	0.04	0.96645
math - shapes	0.0002	0.0005	11958.76	-0.0008	0.0011	0.31	0.75954
math - story	0.0000	0.0005	11922.26	-0.0010	0.0010	-0.08	0.93699
math - ToM	0.0010	0.0005	11955.72	0.0001	0.0020	2.09	0.03631
motor - punish	0.0009	0.0005	11938.83	-0.0001	0.0019	1.82	0.06934
motor - random	0.0017	0.0005	11974.44	0.0007	0.0026	3.39	0.00071
motor - relational	0.0010	0.0005	11963.58	0.0001	0.0020	2.09	0.03685
motor - reward	0.0005	0.0005	11938.83	-0.0005	0.0015	1.00	0.31579
motor - shapes	0.0006	0.0005	11969.31	-0.0003	0.0016	1.26	0.20598
motor - story	0.0004	0.0005	11979.98	-0.0006	0.0014	0.86	0.39190
motor - ToM	0.0015	0.0005	11974.44	0.0006	0.0025	3.08	0.00206
punish - random	0.0008	0.0005	11964.28	-0.0002	0.0017	1.59	0.11101
punish - relational	0.0001	0.0005	11968.54	-0.0008	0.0011	0.30	0.76599
punish - reward	-0.0004	0.0005	11922.26	-0.0014	0.0006	-0.82	0.41211
punish - shapes	-0.0003	0.0005	11959.56	-0.0012	0.0007	-0.54	0.58668
punish - story	-0.0005	0.0005	11982.45	-0.0014	0.0005	-0.92	0.35698
punish - ToM	0.0006	0.0005	11964.28	-0.0003	0.0016	1.29	0.19876
random - relational	-0.0006	0.0005	11946.69	-0.0016	0.0003	-1.28	0.20120
random - reward	-0.0012	0.0005	11964.28	-0.0021	-0.0002	-2.41	0.01599
random - shapes	-0.0010	0.0005	11938.04	-0.0020	-0.0001	-2.13	0.03328
random - story	-0.0012	0.0005	11955.72	-0.0022	-0.0003	-2.48	0.01332
random - ToM	-0.0002	0.0005	11922.26	-0.0011	0.0008	-0.31	0.75807
relational - reward	-0.0005	0.0005	11968.54	-0.0015	0.0004	-1.11	0.26884
relational - shapes	-0.0004	0.0005	11938.46	-0.0014	0.0006	-0.83	0.40437
relational - story	-0.0006	0.0005	11947.83	-0.0016	0.0004	-1.20	0.22913
relational - ToM	0.0005	0.0005	11946.69	-0.0005	0.0015	0.97	0.33048
reward - shapes	0.0001	0.0005	11959.56	-0.0008	0.0011	0.27	0.78671
reward - story	-0.0001	0.0005	11982.45	-0.0010	0.0009	-0.12	0.90236
reward - ToM	0.0010	0.0005	11964.28	0.0001	0.0020	2.10	0.03568
shapes - story	-0.0002	0.0005	11958.76	-0.0012	0.0008	-0.39	0.69923
shapes - ToM	0.0009	0.0005	11938.04	-0.0001	0.0019	1.82	0.06853
story - ToM	0.0011	0.0005	11955.72	0.0001	0.0021	2.17	0.02972

Supplementary Table 8.5 Agreeableness* task condition interactions along D2 (motor-visual)

contrast	β	SE	df	CL lower	CL upper	t.ratio	p.value
0back - 2back	-0.0001	0.0005	11922.26	-0.0010	0.0009	-0.10	0.91781
0back - faces	0.0003	0.0005	11956.14	-0.0006	0.0013	0.67	0.50312
0back - match	0.0008	0.0005	11961.98	-0.0002	0.0017	1.52	0.12859
0back - math	0.0014	0.0005	11989.53	0.0004	0.0024	2.74	0.00611
0back - motor	0.0022	0.0005	11937.59	0.0012	0.0032	4.41	0.00001
0back - punish	0.0007	0.0005	11936.29	-0.0003	0.0017	1.43	0.15328
0back - random	0.0003	0.0005	11968.64	-0.0007	0.0013	0.64	0.52122
0back - relational	0.0004	0.0005	11961.98	-0.0006	0.0013	0.73	0.46271
0back - reward	0.0006	0.0005	11936.29	-0.0004	0.0016	1.19	0.23517
0back - shapes	0.0008	0.0005	11956.14	-0.0001	0.0018	1.69	0.09190
0back - story	0.0020	0.0005	11989.53	0.0010	0.0030	3.87	0.00011
0back - ToM	0.0011	0.0005	11968.64	0.0002	0.0021	2.32	0.02028
2back - faces	0.0004	0.0005	11956.14	-0.0006	0.0014	0.77	0.44010
2back - match	0.0008	0.0005	11961.98	-0.0002	0.0018	1.62	0.10489
2back - math	0.0015	0.0005	11989.53	0.0005	0.0025	2.84	0.00449
2back - motor	0.0022	0.0005	11937.59	0.0013	0.0032	4.51	0.00001
2back - punish	0.0008	0.0005	11936.30	-0.0002	0.0017	1.53	0.12574
2back - random	0.0004	0.0005	11968.65	-0.0006	0.0013	0.74	0.45674
2back - relational	0.0004	0.0005	11961.98	-0.0006	0.0014	0.84	0.40299
2back - reward	0.0006	0.0005	11936.30	-0.0003	0.0016	1.29	0.19697
2back - shapes	0.0009	0.0005	11956.14	-0.0001	0.0019	1.79	0.07380
2back - story	0.0020	0.0005	11989.53	0.0010	0.0030	3.97	0.00007
2back - ToM	0.0012	0.0005	11968.65	0.0002	0.0022	2.42	0.01536
faces - match	0.0004	0.0005	11934.83	-0.0006	0.0014	0.85	0.39552
faces - math	0.0011	0.0005	11967.26	0.0001	0.0021	2.08	0.03727
faces - motor	0.0019	0.0005	11963.11	0.0009	0.0028	3.72	0.00020
faces - punish	0.0004	0.0005	11953.27	-0.0006	0.0013	0.75	0.45369
faces - random	0.0000	0.0005	11948.29	-0.0010	0.0010	-0.03	0.97595
faces - relational	0.0000	0.0005	11934.83	-0.0009	0.0010	0.07	0.94586
faces - reward	0.0003	0.0005	11953.27	-0.0007	0.0012	0.51	0.61009
faces - shapes	0.0005	0.0005	11922.26	-0.0005	0.0015	1.01	0.31173
faces - story	0.0016	0.0005	11967.26	0.0006	0.0027	3.21	0.00134
faces - ToM	0.0008	0.0005	11948.29	-0.0002	0.0018	1.64	0.10074
match - math	0.0006	0.0005	11962.78	-0.0004	0.0017	1.25	0.21189
match - motor	0.0014	0.0005	11957.91	0.0004	0.0024	2.85	0.00437
match - punish	-0.0001	0.0005	11959.86	-0.0010	0.0009	-0.11	0.91409
match - random	-0.0004	0.0005	11957.22	-0.0014	0.0005	-0.88	0.37787

match - relational	-0.0004	0.0005	11922.26	-0.0014	0.0006	-0.78	0.43624
match - reward	-0.0002	0.0005	11959.86	-0.0011	0.0008	-0.35	0.72937
match - shapes	0.0001	0.0005	11934.83	-0.0009	0.0011	0.16	0.87565
match - story	0.0012	0.0005	11962.78	0.0002	0.0022	2.37	0.01784
match - ToM	0.0004	0.0005	11957.22	-0.0006	0.0014	0.78	0.43495
math - motor	0.0008	0.0005	11986.69	-0.0002	0.0018	1.53	0.12630
math - punish	-0.0007	0.0005	11984.16	-0.0017	0.0003	-1.36	0.17237
math - random	-0.0011	0.0005	11974.75	-0.0021	-0.0001	-2.12	0.03412
math - relational	-0.0010	0.0005	11962.78	-0.0020	0.0000	-2.01	0.04467
math - reward	-0.0008	0.0005	11984.16	-0.0018	0.0002	-1.60	0.11027
math - shapes	-0.0006	0.0005	11967.26	-0.0016	0.0004	-1.10	0.27083
math - story	0.0006	0.0005	11922.26	-0.0005	0.0016	1.10	0.27148
math - ToM	-0.0003	0.0005	11974.75	-0.0013	0.0007	-0.50	0.62022
motor - punish	-0.0015	0.0005	11939.70	-0.0025	-0.0005	-2.99	0.00279
motor - random	-0.0019	0.0005	11979.36	-0.0028	-0.0009	-3.76	0.00017
motor - relational	-0.0018	0.0005	11957.91	-0.0028	-0.0008	-3.63	0.00028
motor - reward	-0.0016	0.0005	11939.70	-0.0026	-0.0006	-3.23	0.00124
motor - shapes	-0.0013	0.0005	11963.11	-0.0023	-0.0004	-2.71	0.00681
motor - story	-0.0002	0.0005	11986.69	-0.0012	0.0008	-0.40	0.68618
motor - ToM	-0.0010	0.0005	11979.36	-0.0020	-0.0001	-2.09	0.03681
punish - random	-0.0004	0.0005	11967.54	-0.0014	0.0006	-0.78	0.43428
punish - relational	-0.0003	0.0005	11959.86	-0.0013	0.0006	-0.68	0.49830
punish - reward	-0.0001	0.0005	11922.26	-0.0011	0.0008	-0.24	0.80957
punish - shapes	0.0001	0.0005	11953.27	-0.0008	0.0011	0.27	0.78992
punish - story	0.0013	0.0005	11984.16	0.0003	0.0023	2.50	0.01260
punish - ToM	0.0004	0.0005	11967.54	-0.0005	0.0014	0.90	0.36952
random - relational	0.0000	0.0005	11957.22	-0.0009	0.0010	0.10	0.92189
random - reward	0.0003	0.0005	11967.54	-0.0007	0.0012	0.54	0.58795
random - shapes	0.0005	0.0005	11948.29	-0.0005	0.0015	1.04	0.29636
random - story	0.0017	0.0005	11974.75	0.0007	0.0027	3.25	0.00117
random - ToM	0.0008	0.0005	11922.26	-0.0001	0.0018	1.68	0.09316
relational - reward	0.0002	0.0005	11959.86	-0.0008	0.0012	0.44	0.66060
relational - shapes	0.0005	0.0005	11934.83	-0.0005	0.0015	0.94	0.34812
relational - story	0.0016	0.0005	11962.78	0.0006	0.0026	3.13	0.00176
relational - ToM	0.0008	0.0005	11957.22	-0.0002	0.0018	1.56	0.11771
reward - shapes	0.0003	0.0005	11953.27	-0.0007	0.0012	0.51	0.61303
reward - story	0.0014	0.0005	11984.16	0.0004	0.0024	2.73	0.00639
reward - ToM	0.0006	0.0005	11967.54	-0.0004	0.0015	1.14	0.25535
shapes - story	0.0011	0.0005	11967.26	0.0001	0.0021	2.23	0.02598
shapes - ToM	0.0003	0.0005	11948.29	-0.0007	0.0013	0.63	0.53052
story - ToM	-0.0008	0.0005	11974.75	-0.0018	0.0002	-1.62	0.10424

Supplementary Table 9.1 Neuroticism* task condition interactions along D3 (DMN- FPN)

contrast	β	SE	df	CL_{lower}	CL_{upper}	t.ratio	p.value
0back - 2back	0.0012	0.0005	11937.12	0.0003	0.0021	2.61	0.00903
0back - faces	0.0007	0.0005	11991.40	-0.0003	0.0016	1.43	0.15386
0back - match	0.0005	0.0005	11995.39	-0.0004	0.0015	1.09	0.27660
0back - math	0.0011	0.0005	12006.01	0.0002	0.0021	2.32	0.02061
0back - motor	0.0008	0.0005	11957.22	-0.0001	0.0017	1.70	0.08895
0back - punish	0.0001	0.0005	11959.99	-0.0009	0.0010	0.13	0.89661
0back - random	0.0015	0.0005	11997.00	0.0006	0.0024	3.16	0.00156
0back - relational	0.0008	0.0005	11995.39	-0.0001	0.0018	1.77	0.07730
0back - reward	0.0001	0.0005	11959.99	-0.0008	0.0010	0.15	0.87796
0back - shapes	0.0007	0.0005	11991.40	-0.0002	0.0016	1.51	0.12998
0back - story	0.0001	0.0005	12006.01	-0.0009	0.0010	0.18	0.85639
0back - ToM	0.0005	0.0005	11997.00	-0.0004	0.0015	1.11	0.26684
2back - faces	-0.0005	0.0005	11991.41	-0.0015	0.0004	-1.16	0.24695
2back - match	-0.0007	0.0005	11995.39	-0.0016	0.0002	-1.48	0.13812
2back - math	-0.0001	0.0005	12006.01	-0.0011	0.0008	-0.22	0.82942
2back - motor	-0.0004	0.0005	11957.23	-0.0013	0.0005	-0.90	0.36961
2back - punish	-0.0012	0.0005	11959.99	-0.0021	-0.0002	-2.48	0.01317
2back - random	0.0003	0.0005	11997.01	-0.0007	0.0012	0.57	0.56663
2back - relational	-0.0004	0.0005	11995.39	-0.0013	0.0006	-0.80	0.42127
2back - reward	-0.0012	0.0005	11959.99	-0.0021	-0.0002	-2.46	0.01406
2back - shapes	-0.0005	0.0005	11991.41	-0.0014	0.0004	-1.07	0.28479
2back - story	-0.0011	0.0005	12006.01	-0.0021	-0.0002	-2.35	0.01880
2back - ToM	-0.0007	0.0005	11997.01	-0.0016	0.0002	-1.48	0.13867
faces - match	-0.0002	0.0005	11951.24	-0.0011	0.0008	-0.33	0.74243
faces - math	0.0004	0.0005	11975.56	-0.0005	0.0014	0.91	0.36177
faces - motor	0.0001	0.0005	12000.06	-0.0008	0.0011	0.26	0.79149
faces - punish	-0.0006	0.0005	11989.64	-0.0015	0.0003	-1.30	0.19440
faces - random	0.0008	0.0005	11954.03	-0.0001	0.0018	1.72	0.08510
faces - relational	0.0002	0.0005	11951.24	-0.0008	0.0011	0.35	0.72979
faces - reward	-0.0006	0.0005	11989.64	-0.0015	0.0003	-1.27	0.20256
faces - shapes	0.0000	0.0005	11937.12	-0.0009	0.0010	0.09	0.93019
faces - story	-0.0006	0.0005	11975.56	-0.0015	0.0004	-1.21	0.22740
faces - ToM	-0.0002	0.0005	11954.03	-0.0011	0.0008	-0.32	0.75088
match - math	0.0006	0.0005	11968.70	-0.0004	0.0016	1.23	0.21870
match - motor	0.0003	0.0005	11996.99	-0.0007	0.0012	0.59	0.55340
match - punish	-0.0005	0.0005	11999.00	-0.0014	0.0005	-0.96	0.33707
match - random	0.0010	0.0005	11962.35	0.0000	0.0019	2.04	0.04112
match - relational	0.0003	0.0005	11937.12	-0.0006	0.0013	0.67	0.50205
match - reward	-0.0004	0.0005	11999.00	-0.0014	0.0005	-0.94	0.34890

match - shapes	0.0002	0.0005	11951.24	-0.0007	0.0011	0.42	0.67757
match - story	-0.0004	0.0005	11968.70	-0.0014	0.0005	-0.88	0.37895
match - ToM	0.0000	0.0005	11962.35	-0.0009	0.0009	0.01	0.98913
math - motor	-0.0003	0.0005	12011.20	-0.0013	0.0006	-0.66	0.51203
math - punish	-0.0011	0.0005	12013.91	-0.0020	-0.0001	-2.19	0.02863
math - random	0.0004	0.0005	11980.23	-0.0006	0.0013	0.77	0.44034
math - relational	-0.0003	0.0005	11968.70	-0.0012	0.0007	-0.57	0.56894
math - reward	-0.0011	0.0005	12013.91	-0.0020	-0.0001	-2.17	0.03033
math - shapes	-0.0004	0.0005	11975.56	-0.0014	0.0006	-0.83	0.40866
math - story	-0.0010	0.0005	11937.12	-0.0020	-0.0001	-2.08	0.03723
math - ToM	-0.0006	0.0005	11980.23	-0.0016	0.0004	-1.23	0.22047
motor - punish	-0.0007	0.0005	11960.17	-0.0017	0.0002	-1.57	0.11605
motor - random	0.0007	0.0005	12007.73	-0.0002	0.0016	1.46	0.14404
motor - relational	0.0000	0.0005	11996.99	-0.0009	0.0010	0.08	0.93370
motor - reward	-0.0007	0.0005	11960.17	-0.0017	0.0002	-1.55	0.12161
motor - shapes	-0.0001	0.0005	12000.06	-0.0010	0.0009	-0.18	0.85982
motor - story	-0.0007	0.0005	12011.20	-0.0017	0.0002	-1.47	0.14166
motor - ToM	-0.0003	0.0005	12007.73	-0.0012	0.0007	-0.58	0.55967
punish - random	0.0014	0.0005	11995.58	0.0005	0.0024	3.04	0.00240
punish - relational	0.0008	0.0005	11999.00	-0.0002	0.0017	1.64	0.10130
punish - reward	0.0000	0.0005	11937.12	-0.0009	0.0009	0.02	0.98115
punish - shapes	0.0007	0.0005	11989.64	-0.0003	0.0016	1.39	0.16579
punish - story	0.0000	0.0005	12013.91	-0.0009	0.0010	0.05	0.95619
punish - ToM	0.0005	0.0005	11995.58	-0.0005	0.0014	0.98	0.32635
random - relational	-0.0007	0.0005	11962.35	-0.0016	0.0003	-1.37	0.17172
random - reward	-0.0014	0.0005	11995.58	-0.0024	-0.0005	-3.01	0.00260
random - shapes	-0.0008	0.0005	11954.03	-0.0017	0.0002	-1.63	0.10225
random - story	-0.0014	0.0005	11980.23	-0.0024	-0.0005	-2.90	0.00378
random - ToM	-0.0010	0.0005	11937.12	-0.0019	0.0000	-2.05	0.04073
relational - reward	-0.0008	0.0005	11999.00	-0.0017	0.0002	-1.62	0.10624
relational - shapes	-0.0001	0.0005	11951.24	-0.0011	0.0008	-0.26	0.79620
relational - story	-0.0008	0.0005	11968.70	-0.0017	0.0002	-1.54	0.12351
relational - ToM	-0.0003	0.0005	11962.35	-0.0013	0.0006	-0.66	0.50792
reward - shapes	0.0006	0.0005	11989.64	-0.0003	0.0016	1.36	0.17305
reward - story	0.0000	0.0005	12013.91	-0.0009	0.0010	0.03	0.97444
reward - ToM	0.0005	0.0005	11995.58	-0.0005	0.0014	0.96	0.33804
shapes - story	-0.0006	0.0005	11975.56	-0.0016	0.0003	-1.29	0.19607
shapes - ToM	-0.0002	0.0005	11954.03	-0.0011	0.0007	-0.41	0.68527
story - ToM	0.0004	0.0005	11980.23	-0.0005	0.0014	0.90	0.36841

Supplementary Table 9.2 Openness* task condition interactions along D3 (DMN- FPN)

contrast	β	SE	df	CL lower	CL upper	t.ratio	p.value
0back - 2back	-0.0015	0.0005	11937.12	-0.0025	-0.0005	-2.99	0.00284
0back - faces	-0.0005	0.0005	11987.74	-0.0015	0.0005	-1.06	0.29013
0back - match	-0.0002	0.0005	11993.24	-0.0012	0.0008	-0.38	0.70488
0back - math	-0.0012	0.0005	12010.45	-0.0022	-0.0002	-2.32	0.02022
0back - motor	-0.0015	0.0005	11960.29	-0.0025	-0.0006	-3.06	0.00218
0back - punish	-0.0014	0.0005	11957.25	-0.0023	-0.0004	-2.73	0.00627
0back - random	-0.0016	0.0005	11988.51	-0.0026	-0.0006	-3.17	0.00153
0back - relational	-0.0009	0.0005	11993.24	-0.0019	0.0001	-1.75	0.08007
0back - reward	-0.0018	0.0005	11957.25	-0.0027	-0.0008	-3.58	0.00035
0back - shapes	-0.0013	0.0005	11987.74	-0.0023	-0.0003	-2.59	0.00963
0back - story	0.0006	0.0005	12010.45	-0.0004	0.0016	1.10	0.27095
0back - ToM	0.0000	0.0005	11988.51	-0.0010	0.0010	0.03	0.97679
2back - faces	0.0010	0.0005	11987.74	0.0000	0.0019	1.90	0.05806
2back - match	0.0013	0.0005	11993.24	0.0003	0.0023	2.56	0.01054
2back - math	0.0003	0.0005	12010.45	-0.0007	0.0013	0.56	0.57465
2back - motor	0.0000	0.0005	11960.29	-0.0010	0.0009	-0.10	0.92103
2back - punish	0.0001	0.0005	11957.25	-0.0008	0.0011	0.26	0.79641
2back - random	-0.0001	0.0005	11988.51	-0.0011	0.0009	-0.21	0.83547
2back - relational	0.0006	0.0005	11993.24	-0.0004	0.0016	1.19	0.23551
2back - reward	-0.0003	0.0005	11957.25	-0.0013	0.0007	-0.59	0.55798
2back - shapes	0.0002	0.0005	11987.74	-0.0008	0.0012	0.36	0.71588
2back - story	0.0021	0.0005	12010.45	0.0010	0.0031	3.98	0.00007
2back - ToM	0.0015	0.0005	11988.51	0.0005	0.0025	2.99	0.00278
faces - match	0.0003	0.0005	11959.50	-0.0007	0.0013	0.67	0.50396
faces - math	-0.0007	0.0005	11984.64	-0.0017	0.0004	-1.28	0.20047
faces - motor	-0.0010	0.0005	11998.97	-0.0020	0.0000	-1.98	0.04745
faces - punish	-0.0008	0.0005	11988.13	-0.0018	0.0002	-1.65	0.09985
faces - random	-0.0011	0.0005	11949.12	-0.0020	-0.0001	-2.09	0.03631
faces - relational	-0.0004	0.0005	11959.50	-0.0014	0.0006	-0.69	0.48807
faces - reward	-0.0012	0.0005	11988.13	-0.0022	-0.0003	-2.48	0.01311
faces - shapes	-0.0008	0.0005	11937.12	-0.0018	0.0002	-1.52	0.12822
faces - story	0.0011	0.0005	11984.64	0.0001	0.0021	2.12	0.03416
faces - ToM	0.0005	0.0005	11949.12	-0.0004	0.0015	1.08	0.27913
match - math	-0.0010	0.0005	11972.06	-0.0020	0.0000	-1.93	0.05392
match - motor	-0.0013	0.0005	11995.66	-0.0023	-0.0003	-2.64	0.00826
match - punish	-0.0012	0.0005	12001.58	-0.0022	-0.0002	-2.31	0.02086
match - random	-0.0014	0.0005	11964.58	-0.0024	-0.0004	-2.75	0.00594
match - relational	-0.0007	0.0005	11937.12	-0.0017	0.0003	-1.36	0.17516
match - reward	-0.0016	0.0005	12001.58	-0.0026	-0.0006	-3.14	0.00169

match - shapes	-0.0011	0.0005	11959.50	-0.0021	-0.0001	-2.18	0.02927
match - story	0.0008	0.0005	11972.06	-0.0003	0.0018	1.46	0.14556
match - ToM	0.0002	0.0005	11964.58	-0.0008	0.0012	0.41	0.68494
math - motor	-0.0003	0.0005	12009.06	-0.0014	0.0007	-0.65	0.51294
math - punish	-0.0002	0.0005	12017.39	-0.0012	0.0008	-0.31	0.75402
math - random	-0.0004	0.0005	11982.40	-0.0014	0.0006	-0.76	0.44720
math - relational	0.0003	0.0005	11972.06	-0.0007	0.0013	0.60	0.55078
math - reward	-0.0006	0.0005	12017.39	-0.0016	0.0004	-1.13	0.25899
math - shapes	-0.0001	0.0005	11984.64	-0.0011	0.0009	-0.20	0.83811
math - story	0.0018	0.0005	11937.12	0.0007	0.0028	3.34	0.00086
math - ToM	0.0012	0.0005	11982.40	0.0002	0.0022	2.34	0.01926
motor - punish	0.0002	0.0005	11961.01	-0.0008	0.0012	0.36	0.72190
motor - random	-0.0001	0.0005	11997.48	-0.0010	0.0009	-0.11	0.91397
motor - relational	0.0006	0.0005	11995.66	-0.0003	0.0016	1.28	0.20143
motor - reward	-0.0002	0.0005	11961.01	-0.0012	0.0007	-0.48	0.62908
motor - shapes	0.0002	0.0005	11998.97	-0.0008	0.0012	0.46	0.64553
motor - story	0.0021	0.0005	12009.06	0.0011	0.0031	4.06	0.00005
motor - ToM	0.0015	0.0005	11997.48	0.0006	0.0025	3.07	0.00213
punish - random	-0.0002	0.0005	11982.77	-0.0012	0.0007	-0.46	0.64221
punish - relational	0.0005	0.0005	12001.58	-0.0005	0.0015	0.94	0.34952
punish - reward	-0.0004	0.0005	11937.12	-0.0014	0.0006	-0.85	0.39702
punish - shapes	0.0001	0.0005	11988.13	-0.0009	0.0010	0.11	0.91270
punish - story	0.0019	0.0005	12017.39	0.0009	0.0029	3.75	0.00018
punish - ToM	0.0014	0.0005	11982.77	0.0004	0.0024	2.74	0.00607
random - relational	0.0007	0.0005	11964.58	-0.0003	0.0017	1.39	0.16580
random - reward	-0.0002	0.0005	11982.77	-0.0012	0.0008	-0.37	0.70872
random - shapes	0.0003	0.0005	11949.12	-0.0007	0.0013	0.57	0.56961
random - story	0.0022	0.0005	11982.40	0.0011	0.0032	4.17	0.00003
random - ToM	0.0016	0.0005	11937.12	0.0006	0.0026	3.19	0.00144
relational - reward	-0.0009	0.0005	12001.58	-0.0019	0.0001	-1.77	0.07741
relational - shapes	-0.0004	0.0005	11959.50	-0.0014	0.0006	-0.82	0.41315
relational - story	0.0015	0.0005	11972.06	0.0004	0.0025	2.79	0.00534
relational - ToM	0.0009	0.0005	11964.58	-0.0001	0.0019	1.77	0.07655
reward - shapes	0.0005	0.0005	11988.13	-0.0005	0.0015	0.95	0.34461
reward - story	0.0023	0.0005	12017.39	0.0013	0.0034	4.56	0.00001
reward - ToM	0.0018	0.0005	11982.77	0.0008	0.0028	3.58	0.00034
shapes - story	0.0019	0.0005	11984.64	0.0009	0.0029	3.60	0.00032
shapes - ToM	0.0013	0.0005	11949.12	0.0003	0.0023	2.61	0.00914
story - ToM	-0.0006	0.0005	11982.40	-0.0016	0.0005	-1.07	0.28556

Supplementary Table 9.3 Conscientiousness* task condition interactions along D3 (DMN-FPN)

contrast	β	SE	df	CL_{lower}	CL_{upper}	t.ratio	p.value
0back - 2back	0.0016	0.0006	11937.12	0.0005	0.0027	2.81	0.00501
0back - faces	0.0020	0.0006	12000.84	0.0009	0.0032	3.53	0.00042
0back - match	0.0011	0.0006	12005.49	-0.0001	0.0022	1.87	0.06105
0back - math	0.0013	0.0006	12024.37	0.0002	0.0025	2.26	0.02388
0back - motor	0.0025	0.0006	11953.33	0.0013	0.0036	4.30	0.00002
0back - punish	0.0006	0.0006	11960.85	-0.0005	0.0017	1.10	0.27291
0back - random	0.0015	0.0006	12008.75	0.0004	0.0026	2.58	0.00993
0back - relational	0.0014	0.0006	12005.49	0.0003	0.0026	2.45	0.01419
0back - reward	0.0002	0.0006	11960.85	-0.0009	0.0014	0.41	0.67858
0back - shapes	0.0022	0.0006	12000.84	0.0011	0.0033	3.82	0.00013
0back - story	0.0005	0.0006	12024.37	-0.0007	0.0016	0.77	0.43853
0back - ToM	0.0007	0.0006	12008.75	-0.0004	0.0018	1.22	0.22187
2back - faces	0.0004	0.0006	12000.84	-0.0007	0.0016	0.75	0.45403
2back - match	-0.0005	0.0006	12005.49	-0.0016	0.0006	-0.89	0.37443
2back - math	-0.0003	0.0006	12024.37	-0.0014	0.0009	-0.46	0.64722
2back - motor	0.0009	0.0006	11953.33	-0.0003	0.0020	1.51	0.13072
2back - punish	-0.0010	0.0006	11960.85	-0.0021	0.0001	-1.72	0.08605
2back - random	-0.0001	0.0006	12008.75	-0.0012	0.0010	-0.20	0.84318
2back - relational	-0.0002	0.0006	12005.49	-0.0013	0.0010	-0.31	0.75748
2back - reward	-0.0014	0.0006	11960.85	-0.0025	-0.0002	-2.40	0.01647
2back - shapes	0.0006	0.0006	12000.84	-0.0005	0.0017	1.04	0.29624
2back - story	-0.0011	0.0006	12024.37	-0.0023	0.0000	-1.94	0.05212
2back - ToM	-0.0009	0.0006	12008.75	-0.0020	0.0002	-1.55	0.11998
faces - match	-0.0009	0.0006	11950.73	-0.0021	0.0002	-1.62	0.10464
faces - math	-0.0007	0.0006	11977.40	-0.0019	0.0005	-1.18	0.23687
faces - motor	0.0004	0.0006	12007.61	-0.0007	0.0016	0.75	0.45231
faces - punish	-0.0014	0.0006	11995.27	-0.0025	-0.0003	-2.45	0.01423
faces - random	-0.0005	0.0006	11956.08	-0.0017	0.0006	-0.94	0.34699
faces - relational	-0.0006	0.0006	11950.73	-0.0018	0.0005	-1.05	0.29516
faces - reward	-0.0018	0.0006	11995.27	-0.0029	-0.0007	-3.13	0.00177
faces - shapes	0.0002	0.0006	11937.12	-0.0010	0.0013	0.29	0.76865
faces - story	-0.0016	0.0006	11977.40	-0.0027	-0.0004	-2.66	0.00786
faces - ToM	-0.0013	0.0006	11956.08	-0.0025	-0.0002	-2.29	0.02208
match - math	0.0002	0.0006	11975.22	-0.0009	0.0014	0.41	0.68061
match - motor	0.0014	0.0006	12004.16	0.0002	0.0025	2.37	0.01771
match - punish	-0.0005	0.0006	12002.80	-0.0016	0.0007	-0.80	0.42402
match - random	0.0004	0.0006	11966.06	-0.0007	0.0015	0.69	0.49215

match - relational	0.0003	0.0006	11937.12	-0.0008	0.0015	0.57	0.56650
match - reward	-0.0008	0.0006	12002.80	-0.0020	0.0003	-1.47	0.14130
match - shapes	0.0011	0.0006	11950.73	0.0000	0.0023	1.92	0.05548
match - story	-0.0006	0.0006	11975.22	-0.0018	0.0005	-1.06	0.29073
match - ToM	-0.0004	0.0006	11966.06	-0.0015	0.0008	-0.65	0.51314
math - motor	0.0011	0.0006	12014.87	0.0000	0.0023	1.92	0.05478
math - punish	-0.0007	0.0006	12020.13	-0.0019	0.0004	-1.20	0.22856
math - random	0.0002	0.0006	11987.21	-0.0010	0.0013	0.26	0.79305
math - relational	0.0001	0.0006	11975.22	-0.0011	0.0013	0.15	0.87909
math - reward	-0.0011	0.0006	12020.13	-0.0022	0.0001	-1.86	0.06222
math - shapes	0.0009	0.0006	11977.40	-0.0003	0.0020	1.47	0.14143
math - story	-0.0009	0.0006	11937.12	-0.0021	0.0003	-1.45	0.14710
math - ToM	-0.0006	0.0006	11987.21	-0.0018	0.0005	-1.06	0.29048
motor - punish	-0.0018	0.0006	11966.79	-0.0030	-0.0007	-3.22	0.00127
motor - random	-0.0010	0.0006	12015.48	-0.0021	0.0002	-1.69	0.09057
motor - relational	-0.0010	0.0006	12004.16	-0.0022	0.0001	-1.80	0.07260
motor - reward	-0.0022	0.0006	11966.79	-0.0034	-0.0011	-3.90	0.00010
motor - shapes	-0.0003	0.0006	12007.61	-0.0014	0.0009	-0.46	0.64743
motor - story	-0.0020	0.0006	12014.87	-0.0032	-0.0009	-3.40	0.00068
motor - ToM	-0.0018	0.0006	12015.48	-0.0029	-0.0006	-3.04	0.00235
punish - random	0.0009	0.0006	11996.42	-0.0003	0.0020	1.50	0.13318
punish - relational	0.0008	0.0006	12002.80	-0.0003	0.0019	1.38	0.16739
punish - reward	-0.0004	0.0006	11937.12	-0.0015	0.0007	-0.68	0.49354
punish - shapes	0.0016	0.0006	11995.27	0.0005	0.0027	2.75	0.00600
punish - story	-0.0002	0.0006	12020.13	-0.0013	0.0010	-0.29	0.77555
punish - ToM	0.0001	0.0006	11996.42	-0.0010	0.0012	0.14	0.88871
random - relational	-0.0001	0.0006	11966.06	-0.0012	0.0011	-0.11	0.91139
random - reward	-0.0012	0.0006	11996.42	-0.0024	-0.0001	-2.18	0.02948
random - shapes	0.0007	0.0006	11956.08	-0.0004	0.0019	1.23	0.21707
random - story	-0.0010	0.0006	11987.21	-0.0022	0.0001	-1.74	0.08233
random - ToM	-0.0008	0.0006	11937.12	-0.0019	0.0004	-1.35	0.17713
relational - reward	-0.0012	0.0006	12002.80	-0.0023	-0.0001	-2.05	0.04016
relational - shapes	0.0008	0.0006	11950.73	-0.0004	0.0019	1.34	0.18050
relational - story	-0.0010	0.0006	11975.22	-0.0021	0.0002	-1.62	0.10519
relational - ToM	-0.0007	0.0006	11966.06	-0.0019	0.0004	-1.23	0.21887
reward - shapes	0.0020	0.0006	11995.27	0.0008	0.0031	3.42	0.00062
reward - story	0.0002	0.0006	12020.13	-0.0009	0.0014	0.38	0.70721
reward - ToM	0.0005	0.0006	11996.42	-0.0007	0.0016	0.82	0.41485
shapes - story	-0.0017	0.0006	11977.40	-0.0029	-0.0006	-2.95	0.00322
shapes - ToM	-0.0015	0.0006	11956.08	-0.0026	-0.0004	-2.58	0.00980
story - ToM	0.0002	0.0006	11987.21	-0.0009	0.0014	0.42	0.67595

Supplementary Table 9.4 Extraversion* task condition interactions along D3 (DMN- FPN)

contrast	β	SE	df	CL $_{lower}$	CL $_{upper}$	t.ratio	p.value
0back - 2back	0.0004	0.0006	11937.12	-0.0007	0.0015	0.75	0.45031
0back - faces	-0.0002	0.0006	11981.63	-0.0013	0.0009	-0.37	0.71416
0back - match	-0.0004	0.0006	11992.15	-0.0015	0.0007	-0.77	0.44284
0back - math	0.0001	0.0006	12007.53	-0.0010	0.0012	0.17	0.86594
0back - motor	0.0004	0.0006	11960.83	-0.0007	0.0015	0.79	0.42668
0back - punish	-0.0011	0.0006	11950.21	-0.0022	0.0000	-1.95	0.05174
0back - random	0.0002	0.0006	11982.70	-0.0009	0.0013	0.34	0.73319
0back - relational	-0.0002	0.0006	11992.15	-0.0014	0.0009	-0.44	0.65802
0back - reward	-0.0008	0.0006	11950.21	-0.0019	0.0002	-1.53	0.12625
0back - shapes	-0.0010	0.0006	11981.63	-0.0021	0.0000	-1.88	0.06026
0back - story	-0.0001	0.0006	12007.53	-0.0012	0.0010	-0.15	0.87894
0back - ToM	-0.0006	0.0006	11982.70	-0.0017	0.0005	-1.13	0.25676
2back - faces	-0.0006	0.0006	11981.63	-0.0017	0.0005	-1.12	0.26421
2back - match	-0.0008	0.0006	11992.15	-0.0020	0.0003	-1.51	0.13053
2back - math	-0.0003	0.0006	12007.53	-0.0014	0.0008	-0.57	0.57071
2back - motor	0.0000	0.0006	11960.83	-0.0011	0.0011	0.05	0.96353
2back - punish	-0.0015	0.0006	11950.21	-0.0026	-0.0004	-2.70	0.00693
2back - random	-0.0002	0.0006	11982.70	-0.0013	0.0009	-0.41	0.68130
2back - relational	-0.0007	0.0006	11992.15	-0.0018	0.0004	-1.19	0.23511
2back - reward	-0.0013	0.0006	11950.21	-0.0024	-0.0002	-2.28	0.02236
2back - shapes	-0.0015	0.0006	11981.63	-0.0026	-0.0004	-2.63	0.00857
2back - story	-0.0005	0.0006	12007.53	-0.0016	0.0006	-0.89	0.37447
2back - ToM	-0.0011	0.0006	11982.70	-0.0021	0.0000	-1.89	0.05935
faces - match	-0.0002	0.0006	11955.59	-0.0013	0.0009	-0.40	0.68717
faces - math	0.0003	0.0006	11978.58	-0.0008	0.0014	0.53	0.59860
faces - motor	0.0006	0.0006	11989.43	-0.0005	0.0017	1.15	0.24833
faces - punish	-0.0009	0.0006	11978.19	-0.0020	0.0002	-1.57	0.11687
faces - random	0.0004	0.0006	11954.02	-0.0007	0.0015	0.71	0.48053
faces - relational	0.0000	0.0006	11955.59	-0.0011	0.0011	-0.08	0.93714
faces - reward	-0.0006	0.0006	11978.19	-0.0017	0.0004	-1.15	0.24849
faces - shapes	-0.0008	0.0006	11937.12	-0.0019	0.0003	-1.51	0.13137
faces - story	0.0001	0.0006	11978.58	-0.0010	0.0012	0.21	0.83657
faces - ToM	-0.0004	0.0006	11954.02	-0.0015	0.0007	-0.76	0.44449
match - math	0.0005	0.0006	11966.42	-0.0006	0.0017	0.92	0.35834
match - motor	0.0009	0.0006	11982.91	-0.0002	0.0020	1.55	0.12157
match - punish	-0.0006	0.0006	11988.62	-0.0017	0.0005	-1.15	0.24930
match - random	0.0006	0.0006	11964.16	-0.0005	0.0017	1.10	0.26982
match - relational	0.0002	0.0006	11937.12	-0.0009	0.0013	0.32	0.74753
match - reward	-0.0004	0.0006	11988.62	-0.0015	0.0007	-0.74	0.45864

match - shapes	-0.0006	0.0006	11955.59	-0.0017	0.0005	-1.09	0.27373
match - story	0.0003	0.0006	11966.42	-0.0008	0.0015	0.60	0.54826
match - ToM	-0.0002	0.0006	11964.16	-0.0013	0.0009	-0.36	0.72203
math - motor	0.0003	0.0006	12001.32	-0.0008	0.0015	0.61	0.54300
math - punish	-0.0012	0.0006	12004.38	-0.0023	-0.0001	-2.07	0.03878
math - random	0.0001	0.0006	11974.73	-0.0010	0.0012	0.16	0.86917
math - relational	-0.0003	0.0006	11966.42	-0.0015	0.0008	-0.60	0.54810
math - reward	-0.0009	0.0006	12004.38	-0.0021	0.0002	-1.66	0.09682
math - shapes	-0.0011	0.0006	11978.58	-0.0023	0.0000	-2.01	0.04493
math - story	-0.0002	0.0006	11937.12	-0.0013	0.0010	-0.32	0.75260
math - ToM	-0.0007	0.0006	11974.73	-0.0018	0.0004	-1.28	0.20143
motor - punish	-0.0015	0.0006	11956.03	-0.0026	-0.0004	-2.73	0.00638
motor - random	-0.0003	0.0006	11994.21	-0.0014	0.0008	-0.45	0.65027
motor - relational	-0.0007	0.0006	11982.91	-0.0018	0.0004	-1.23	0.22042
motor - reward	-0.0013	0.0006	11956.03	-0.0024	-0.0002	-2.31	0.02065
motor - shapes	-0.0015	0.0006	11989.43	-0.0026	-0.0004	-2.66	0.00789
motor - story	-0.0005	0.0006	12001.32	-0.0017	0.0006	-0.93	0.35367
motor - ToM	-0.0011	0.0006	11994.21	-0.0022	0.0000	-1.92	0.05511
punish - random	0.0013	0.0006	11982.70	0.0002	0.0024	2.28	0.02266
punish - relational	0.0008	0.0006	11988.62	-0.0003	0.0019	1.48	0.13963
punish - reward	0.0002	0.0006	11937.12	-0.0009	0.0013	0.42	0.67666
punish - shapes	0.0000	0.0006	11978.19	-0.0011	0.0011	0.05	0.95744
punish - story	0.0010	0.0006	12004.38	-0.0001	0.0021	1.75	0.08099
punish - ToM	0.0004	0.0006	11982.70	-0.0006	0.0015	0.80	0.42220
random - relational	-0.0004	0.0006	11964.16	-0.0015	0.0007	-0.78	0.43585
random - reward	-0.0010	0.0006	11982.70	-0.0021	0.0001	-1.86	0.06225
random - shapes	-0.0012	0.0006	11954.02	-0.0023	-0.0001	-2.22	0.02671
random - story	-0.0003	0.0006	11974.73	-0.0014	0.0008	-0.49	0.62737
random - ToM	-0.0008	0.0006	11937.12	-0.0019	0.0003	-1.47	0.14064
relational - reward	-0.0006	0.0006	11988.62	-0.0017	0.0005	-1.07	0.28631
relational - shapes	-0.0008	0.0006	11955.59	-0.0019	0.0003	-1.42	0.15610
relational - story	0.0002	0.0006	11966.42	-0.0010	0.0013	0.28	0.77760
relational - ToM	-0.0004	0.0006	11964.16	-0.0015	0.0007	-0.68	0.49650
reward - shapes	-0.0002	0.0006	11978.19	-0.0013	0.0009	-0.36	0.71833
reward - story	0.0008	0.0006	12004.38	-0.0004	0.0019	1.34	0.18058
reward - ToM	0.0002	0.0006	11982.70	-0.0009	0.0013	0.39	0.69811
shapes - story	0.0010	0.0006	11978.58	-0.0002	0.0021	1.69	0.09194
shapes - ToM	0.0004	0.0006	11954.02	-0.0007	0.0015	0.75	0.45579
story - ToM	-0.0005	0.0006	11974.73	-0.0017	0.0006	-0.96	0.33868

Supplementary Table 9.5 Agreeableness* task condition interactions along D3 (DMN- FPN)

contrast	β	SE	df	CL_{lower}	CL_{upper}	t.ratio	p.value
0back - 2back	-0.0004	0.0006	11937.12	-0.0015	0.0007	-0.74	0.45790
0back - faces	-0.0011	0.0006	11974.44	-0.0023	0.0000	-2.04	0.04178
0back - match	-0.0009	0.0006	11981.03	-0.0020	0.0003	-1.51	0.13212
0back - math	-0.0007	0.0006	12012.93	-0.0018	0.0005	-1.13	0.26029
0back - motor	-0.0015	0.0006	11954.40	-0.0026	-0.0004	-2.74	0.00618
0back - punish	-0.0005	0.0006	11952.55	-0.0016	0.0006	-0.98	0.32907
0back - random	-0.0002	0.0006	11986.08	-0.0013	0.0009	-0.32	0.75025
0back - relational	-0.0011	0.0006	11981.03	-0.0022	0.0001	-1.86	0.06350
0back - reward	-0.0005	0.0006	11952.55	-0.0016	0.0006	-0.95	0.33987
0back - shapes	-0.0008	0.0006	11974.44	-0.0019	0.0003	-1.35	0.17696
0back - story	-0.0005	0.0006	12012.93	-0.0016	0.0006	-0.85	0.39287
0back - ToM	0.0006	0.0006	11986.08	-0.0005	0.0017	0.99	0.32014
2back - faces	-0.0007	0.0006	11974.44	-0.0018	0.0004	-1.30	0.19397
2back - match	-0.0004	0.0006	11981.03	-0.0015	0.0007	-0.77	0.43972
2back - math	-0.0002	0.0006	12012.93	-0.0014	0.0009	-0.41	0.68164
2back - motor	-0.0011	0.0006	11954.40	-0.0022	0.0000	-2.00	0.04541
2back - punish	-0.0001	0.0006	11952.56	-0.0012	0.0010	-0.23	0.81464
2back - random	0.0002	0.0006	11986.09	-0.0009	0.0013	0.42	0.67373
2back - relational	-0.0006	0.0006	11981.03	-0.0017	0.0005	-1.12	0.26162
2back - reward	-0.0001	0.0006	11952.56	-0.0012	0.0010	-0.21	0.83144
2back - shapes	-0.0003	0.0006	11974.44	-0.0014	0.0008	-0.61	0.53969
2back - story	-0.0001	0.0006	12012.93	-0.0012	0.0011	-0.14	0.88950
2back - ToM	0.0010	0.0006	11986.09	-0.0001	0.0021	1.73	0.08302
faces - match	0.0003	0.0006	11951.28	-0.0008	0.0014	0.52	0.60497
faces - math	0.0005	0.0006	11988.84	-0.0006	0.0016	0.85	0.39700
faces - motor	-0.0004	0.0006	11982.16	-0.0015	0.0007	-0.70	0.48634
faces - punish	0.0006	0.0006	11971.14	-0.0005	0.0017	1.07	0.28656
faces - random	0.0010	0.0006	11964.19	-0.0001	0.0021	1.72	0.08627
faces - relational	0.0001	0.0006	11951.28	-0.0010	0.0012	0.17	0.86580
faces - reward	0.0006	0.0006	11971.14	-0.0005	0.0017	1.09	0.27698
faces - shapes	0.0004	0.0006	11937.12	-0.0007	0.0015	0.68	0.49482
faces - story	0.0007	0.0006	11988.84	-0.0005	0.0018	1.12	0.26399
faces - ToM	0.0017	0.0006	11964.19	0.0006	0.0028	3.02	0.00252
match - math	0.0002	0.0006	11983.72	-0.0009	0.0013	0.34	0.73332
match - motor	-0.0007	0.0006	11976.57	-0.0018	0.0004	-1.21	0.22634
match - punish	0.0003	0.0006	11978.78	-0.0008	0.0014	0.54	0.58874
match - random	0.0007	0.0006	11974.26	-0.0004	0.0018	1.19	0.23493
match - relational	-0.0002	0.0006	11937.12	-0.0013	0.0009	-0.35	0.72871
match - reward	0.0003	0.0006	11978.78	-0.0008	0.0014	0.56	0.57412

match - shapes	0.0001	0.0006	11951.28	-0.0010	0.0012	0.16	0.87151
match - story	0.0004	0.0006	11983.72	-0.0008	0.0015	0.61	0.54215
match - ToM	0.0014	0.0006	11974.26	0.0003	0.0025	2.49	0.01290
math - motor	-0.0009	0.0006	12009.56	-0.0020	0.0003	-1.52	0.12779
math - punish	0.0001	0.0006	12007.02	-0.0010	0.0012	0.18	0.85402
math - random	0.0005	0.0006	11995.59	-0.0007	0.0016	0.82	0.41423
math - relational	-0.0004	0.0006	11983.72	-0.0015	0.0007	-0.68	0.49707
math - reward	0.0001	0.0006	12007.02	-0.0010	0.0013	0.20	0.83772
math - shapes	-0.0001	0.0006	11988.84	-0.0013	0.0010	-0.18	0.85374
math - story	0.0002	0.0006	11937.12	-0.0010	0.0013	0.26	0.79197
math - ToM	0.0012	0.0006	11995.59	0.0001	0.0024	2.08	0.03711
motor - punish	0.0010	0.0006	11956.36	-0.0001	0.0021	1.77	0.07726
motor - random	0.0014	0.0006	11998.01	0.0003	0.0025	2.41	0.01589
motor - relational	0.0005	0.0006	11976.57	-0.0006	0.0016	0.86	0.38878
motor - reward	0.0010	0.0006	11956.36	-0.0001	0.0021	1.79	0.07373
motor - shapes	0.0008	0.0006	11982.16	-0.0003	0.0019	1.38	0.16830
motor - story	0.0010	0.0006	12009.56	-0.0001	0.0022	1.79	0.07302
motor - ToM	0.0021	0.0006	11998.01	0.0010	0.0032	3.72	0.00020
punish - random	0.0004	0.0006	11985.17	-0.0007	0.0015	0.65	0.51277
punish - relational	-0.0005	0.0006	11978.78	-0.0016	0.0006	-0.89	0.37323
punish - reward	0.0000	0.0006	11937.12	-0.0011	0.0011	0.02	0.98278
punish - shapes	-0.0002	0.0006	11971.14	-0.0013	0.0009	-0.38	0.70379
punish - story	0.0001	0.0006	12007.02	-0.0011	0.0012	0.09	0.93047
punish - ToM	0.0011	0.0006	11985.17	0.0000	0.0022	1.97	0.04925
random - relational	-0.0009	0.0006	11974.26	-0.0020	0.0002	-1.54	0.12431
random - reward	-0.0004	0.0006	11985.17	-0.0015	0.0007	-0.63	0.52672
random - shapes	-0.0006	0.0006	11964.19	-0.0017	0.0005	-1.03	0.30251
random - story	-0.0003	0.0006	11995.59	-0.0015	0.0008	-0.55	0.58535
random - ToM	0.0007	0.0006	11937.12	-0.0004	0.0018	1.31	0.18967
relational - reward	0.0005	0.0006	11978.78	-0.0006	0.0016	0.91	0.36189
relational - shapes	0.0003	0.0006	11951.28	-0.0008	0.0014	0.51	0.61004
relational - story	0.0006	0.0006	11983.72	-0.0006	0.0017	0.95	0.34315
relational - ToM	0.0016	0.0006	11974.26	0.0005	0.0027	2.84	0.00458
reward - shapes	-0.0002	0.0006	11971.14	-0.0013	0.0009	-0.40	0.68794
reward - story	0.0000	0.0006	12007.02	-0.0011	0.0012	0.07	0.94704
reward - ToM	0.0011	0.0006	11985.17	0.0000	0.0022	1.95	0.05179
shapes - story	0.0003	0.0006	11988.84	-0.0009	0.0014	0.45	0.64955
shapes - ToM	0.0013	0.0006	11964.19	0.0002	0.0024	2.34	0.01946
story - ToM	0.0011	0.0006	11995.59	-0.0001	0.0022	1.81	0.06972
