# Supplementary material for: Personality traits vary in their association with brain activity across situations
Source: Commun Biol. 2024 Nov 12;7:1498. doi: 10.1038/s42003-024-07061-0 (PMC11557894; doi:10.1038/s42003-024-07061-0)
Supplement: Supplementary file 2 — Reporting Summary [file 42003_2024_7061_MOESM2_ESM.pdf]

nature portfolio

Corresponding author(s): Samyogita Hardikar

Last updated by author(s): 10/08/2024

Reporting Summary

Nature Portfolio wishes to improve the reproducibility of the work that we publish. This form provides structure for consistency and transparency in reporting. For further information on Nature Portfolio policies, see our Editorial Policies and the Editorial Policy Checklist.

Statistics

For all statistical analyses, confirm that the following items are present in the figure legend, table legend, main text, or Methods section.

n/a	Confirmed	
☐	☒	The exact sample size (*n*) for each experimental group/condition, given as a discrete number and unit of measurement
☐	☒	A statement on whether measurements were taken from distinct samples or whether the same sample was measured repeatedly
☐	☒	The statistical test(s) used AND whether they are one- or two-sided<br>*Only common tests should be described solely by name; describe more complex techniques in the Methods section.*
☐	☒	A description of all covariates tested
☐	☒	A description of any assumptions or corrections, such as tests of normality and adjustment for multiple comparisons
☐	☒	A full description of the statistical parameters including central tendency (e.g. means) or other basic estimates (e.g. regression coefficient) AND variation (e.g. standard deviation) or associated estimates of uncertainty (e.g. confidence intervals)
☐	☒	For null hypothesis testing, the test statistic (e.g. *F*, *t*, *r*) with confidence intervals, effect sizes, degrees of freedom and *P* value noted<br>*Give P values as exact values whenever suitable.*
☒	☐	For Bayesian analysis, information on the choice of priors and Markov chain Monte Carlo settings
☒	☐	For hierarchical and complex designs, identification of the appropriate level for tests and full reporting of outcomes
☐	☒	Estimates of effect sizes (e.g. Cohen's *d*, Pearson's *r*), indicating how they were calculated

Our web collection on statistics for biologists contains articles on many of the points above.

Software and code

Policy information about availability of computer code

Data collection	This study did not involve data collection. All data used was collected described and published previously by the Human Connectome Project [HCP; van Essen et al., 2013, Barch et al., 2013].
Data analysis	We used R [R Core Team. R: A language and environment for statistical computing. (2021)] for performing the analyses. All code used for analysis and visualization can be found at https://github.com/samyogita-hardikar/hcp-task-trait

For manuscripts utilizing custom algorithms or software that are central to the research but not yet described in published literature, software must be made available to editors and reviewers. We strongly encourage code deposition in a community repository (e.g. GitHub). See the Nature Portfolio guidelines for submitting code & software for further information.

Data

Policy information about availability of data

All manuscripts must include a data availability statement. This statement should provide the following information, where applicable:

- Accession codes, unique identifiers, or web links for publicly available datasets
- A description of any restrictions on data availability
- For clinical datasets or third party data, please ensure that the statement adheres to our policy

All subject-level data included in the present study are available for download through the HCP s1200 release at https://db.humanconnectome.org/. Sensitive data such as exact age in years are available only to qualified investigators who agree to HCP's Restricted Data Use Terms. Details can be found at https://

www.humanconnectome.org/study/hcp-young-adult/document/restricted-data-usage. Connectivity gradients maps are available for download at: https://www.neuroconnlab.org/data/

Research involving human participants, their data, or biological material

Policy information about studies with human participants or human data. See also policy information about sex, gender (identity/presentation), and sexual orientation and race, ethnicity and racism.

Reporting on sex and gender	We refer to the binary self-report variable "gender" (male/ female) as included in the HCP data, although we acknowledge that this does not capture the complete spectrum of gender identity and expression. Gender was included as a covariate of no interest in the analyses, but the study did not include any gender-specific hypothesis or gender-stratified analyses.
Reporting on race, ethnicity, or other socially relevant groupings	The analyses did not include any constructs related to race, ethnicity or social groupings.
Population characteristics	The HCP sample primarily includes healthy individuals between 18-36 years of age born in Missouri to families that include twins, and who broadly reflect the ethnic and racial composition of the U.S. population as represented in the 2000 decennial census.The HCP excluded sibships with individuals having severe neuro-developmental, neuropsychiatric or neurological disorders, and excluded individuals with illnesses such as diabetes or high blood pressure, twins born prior to 34 weeks gestation and non-twins born prior to 37 weeks gestation. Individuals who are smokers overweight, or have a history of heavy drinking or recreational drug use without symptoms are not exclude. [van Essen et al., 2013].
Recruitment	HCP recruited participants based on data from the Missouri Department of Health and Senior Services Bureau of Vital Records [van Essen et al., 2013].
Ethics oversight	Data acquisition protocols of the Human Connectome Project [van Essen et al., 2013] were approved by the Washington University institutional review board, and all participants provided written informed consent.

Note that full information on the approval of the study protocol must also be provided in the manuscript.

Field-specific reporting

Please select the one below that is the best fit for your research. If you are not sure, read the appropriate sections before making your selection.

☐ Life sciences ☒ Behavioural & social sciences ☐ Ecological, evolutionary & environmental sciences

For a reference copy of the document with all sections, see nature.com/documents/nr-reporting-summary-flat.pdf

Behavioural & social sciences study design

All studies must disclose on these points even when the disclosure is negative.

Study description	This study used quantitative fMRI and self-report data to analyse the associations between personality traits and brain-wide activity patterns across tasks, and the stability of these associations across differences in sample size.
Research sample	We used data from the Human Connectome Project [van Essen et al., 2013], as it is the only publicly available dataset that includes subject level MRi data across seven different task contexts, as well as the "big five" personality traits and demographic information in a large sample (N= 1200). Additionally, we used previously published low-dimentional summaries of the preprocessed dense connetome from the S900 release of the HCP [Margulies et al., 2016]
Sampling strategy	See above.
Data collection	see van Essen et al, 2013
Timing	see van Essen et al, 2013
Data exclusions	We did not apply any exclusion criteria. All HCP subjects with available task fMRI contrast maps were included in the analysis.
Non-participation	n/a
Randomization	No randomization was implemented as the HCP data collection protocols did not involve any experimental groups.

Reporting for specific materials, systems and methods

We require information from authors about some types of materials, experimental systems and methods used in many studies. Here, indicate whether each material, system or method listed is relevant to your study. If you are not sure if a list item applies to your research, read the appropriate section before selecting a response.

Materials & experimental systems

n/a	Involved in the study
☒	☐ Antibodies
☒	☐ Eukaryotic cell lines
☒	☐ Palaeontology and archaeology
☒	☐ Animals and other organisms
☒	☐ Clinical data
☒	☐ Dual use research of concern
☒	☐ Plants

Methods

n/a	Involved in the study
☒	☐ ChIP-seq
☒	☐ Flow cytometry
☐	☒ MRI-based neuroimaging

Plants

Seed stocks

Report on the source of all seed stocks or other plant material used. If applicable, state the seed stock centre and catalogue number. If plant specimens were collected from the field, describe the collection location, date and sampling procedures.

Novel plant genotypes

Describe the methods by which all novel plant genotypes were produced. This includes those generated by transgenic approaches, gene editing, chemical/radiation-based mutagenesis and hybridization. For transgenic lines, describe the transformation method, the number of independent lines analyzed and the generation upon which experiments were performed. For gene-edited lines, describe the editor used, the endogenous sequence targeted for editing, the targeting guide RNA sequence (if applicable) and how the editor was applied.

Authentication

Describe any authentication procedures for each seed stock used or novel genotype generated. Describe any experiments used to assess the effect of a mutation and, where applicable, how potential secondary effects (e.g. second site T-DNA insertions, mosiacism, off-target gene editing) were examined.

Magnetic resonance imaging

Experimental design

Design type

resting state MRI, task-based MRi

Design specifications

Resting-state : 14:33 min * 4 runs
Tasks:
Working Memory: 5:01 min * 2 runs
Gambling: 3:12 min * 2 runs
Motor: 3:34 min * 2 runs
Language: 3:57 min * 2 runs
Social Cognition: 3:27 min * 2 runs
Relational Processing: 2:56 min * 2 runs
Emotion Processing: 2:16 min * 2 runs

Behavioral performance measures

The current analyses did not use any behavioural performance measures from the tasks. rather, the task conditions were use only as experimentally induced "neural states".

Acquisition

Imaging type(s)

Functional MRI

Field strength

3 Tesla

Sequence & imaging parameters

The HCP used an fMRI protocol (both resting-state and task-evoked) on a customized Skyra "Connectome" with the following parametres: Sequence= Gradient-echo EPI, TR= 720 ms, TE= 33.1 ms, flip angle= 52 deg, FOV= 208x180 mm (RO x PE), Matrix = 104x90 (RO x PE), Slice thickness= 2.0 mm; 72 slices; 2.0 mm isotropic voxels, Multiband factor= 8, Echo spacing= 0.58 ms, Bandwidth= 2290 Hz/Px. Phase encoding direction (RL/ LR) alternated between runs.

Area of acquisition

Whole Brain

Diffusion MRI ☐ Used ☒ Not used

Preprocessing

Preprocessing software

Preprocessing pipelines of the HCP resting-state and task-nased fMRI used customized tools from Connectome Workbench, FSL, and Freesurfer. Detailed descriptions of preprocessing steps can be found in the following publications dedicated to:

	s1200 data release [van Essen et al., 2013], HCP preprocessing pipelines [Glasser et al., 2013], HCP task fMRI [Barch et al., 2013] and HCP resting-state fMRI [Smith et al., 2013].
Normalization	Native volume to MNI nonlinear registration described in Glasser et al., 2013.
Normalization template	Initial volume regisrtation: MNI 152; final data: 91,282 standard grayordinate (CIFTI) space (which combines a standard subcortical segmentation in 2 mm MNI space from the Conte69 subjects, and the 32k conte69 mesh from both hemispheres).
Noise and artifact removal	ICA-based X-noisefier (FIX) in FMRIB's [Salimi-Khorshidi et al., 2014].
Volume censoring	No volume censoring was performed.

Statistical modeling & inference

Model type and settings	Each participants first-level contrast maps for each task condtion were projected onto three orthogonal dimensions of brain variation [i.e. first three "connectivity gradients", Margulies et al., 2016] by calculating Spearman rank correlation between each contrast map and each of the three gradients. We used linear mixed models once for each of three dimensions of brain variation, where location of a specific task-condition map on the relevant dimension was the outcome variable, and the task context, each of five dimensions of personality (Neuroticism, Openness to Experience, Conscientiousness, Extraversion, and Agreeableness) and the interactions between each personality dimension and each task condition were the the predictors. Subject ID and family ID were added as random effects, and age, gender, and mean framewise displacement were included as covariates of no interest.
Effect(s) tested	1) main effect of task condtion<br>2) main effect of personality trait<br>3) trait-by-task interaction<br>Effects were identified with F-tests, and significant interactions were followed up with pairwise comparisons between task conditions.

Specify type of analysis: ☒ Whole brain ☐ ROI-based ☐ Both

Statistic type for inference<br>(See Eklund et al. 2016)	Univariate statistics on whole-brain map similarity scores (see above).
Correction	To account for family wise error in these analyses we controlled for the 78 pairwise comparisons between tasks, the five personality traits and the three dimensions of brain variation (78 * 5 * 3 = 1170) using the Bonferroni method (0.05/1170 = 0.00004).

Models & analysis

n/a	Involved in the study
☒	☐ Functional and/or effective connectivity
☒	☐ Graph analysis
☒	☐ Multivariate modeling or predictive analysis
